# Water‐Triggered Direct Air Capture by Strong Organic Bases

**DOI:** 10.1002/cssc.202402685

**Published:** 2025-02-18

**Authors:** Anders Grundtvig Utzon, Ji‐Woong Lee

**Affiliations:** ^1^ Department of Chemistry University of Copenhagen Universitetsparken 5 Copenhagen Ø 2100 Denmark; ^2^ Nanoscience Center University of Copenhagen Universitetsparken 5 Copenhagen Ø 2100 Denmark; ^3^ The Novo Nordisk Foundation CO2 Research Center Gustav Wieds Vej 10C 8000 Aarhus Denmark

**Keywords:** CO_2_, carbon capture, direct air capture, guanidine, water

## Abstract

A key challenge for sorbent‐mediated temperature‐swing direct air capture remains to maximize the difference in CO_2_ loading under capture and regeneration conditions (i. e. the working capacity) while minimizing the thermal energy input required to alternate between the two equilibrium states. Herein, peralkylated guanidines were shown to capture close to 1 molar equivalent (or up 4.5 mol CO_2_/kg absorbent) directly from moist air (>60 % RH) at room temperature and completely release the entire quantity of captured CO_2_ upon co‐evaporation of water at 70 °C affording concentrated CO_2_ with a vapor content of 3 mol% and achieving a high working capacity with minimum temperature swing of 45 °C.

## Graphical abstract

Peralkylated guanidines capture CO₂ directly from moist air and release concentrated CO₂ upon water co‐evaporation with a smalltemperature‐swing of 45 °C. This water‐triggered capture and release mechanism enables a high working capacity of nearly one equivalent CO_2_ per base, offering a promising approach for direct air capture.

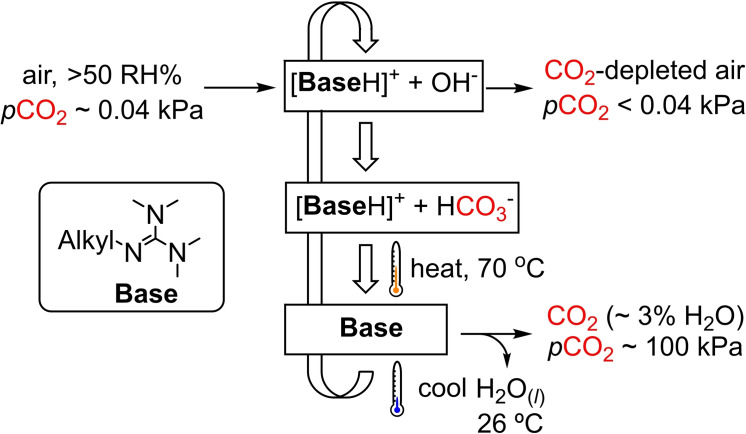

## Introduction

Direct air capture (DAC) with carbon storage (collectively, DACCS) covers a broad spectrum of technologies that aim to remove CO_2_ directly from the atmosphere and store it permanently using chemical or physical processes.[^1^, ^2^, ^3^, ^4^] In principle, DAC involves two stages; (i) a capture stage, where a material/solution (namely sorbent) selectively captures CO_2_ from air thereby affording CO_2_ in a bound state onto/within the sorbent; (ii) a regeneration stage, where pure CO_2_ gas is released from the surface/bulk of the sorbent. The sorbent remains unchanged by the net process while CO_2_ is separated from air, which can then be compressed and stored. As of today, there are a handful of commercial DAC processes in operation and countless more in development.^[5]^ Several technical objectives are required to commercialize an efficient DAC process; the sorbent must be inexpensive, non‐toxic, scalable, it must exhibit fast kinetics and have a high working capacity for prompt and wide deployment.^[6]^

Equilibrium properties of the sorbent are crucial to consider early in development of a DAC system because they dictate whether it is theoretically possible to capture and isolate CO_2_ from air in a cyclic process.^[7]^ These typically include state variables such as *p*CO_2_, temperature and CO_2_ loading of the sorbent under capture and regeneration conditions. A key challenge is to maximize the CO_2_ loading of the sorbent during capture conditions (where *p*CO_2_=0.04 kPa), minimize CO_2_ loading under regeneration conditions (where ideally *p*CO_2_~100 kPa) while minimizing the energy penalty involved in the regeneration stage.^[8]^

In order to regenerate a CO_2_−loaded sorbent post capture stage, it is necessary to impose a stimulus, which favors CO_2_ release to the gas phase. The most common stimuli are temperature‐swings (TS), pressure‐swings (PS) and vacuum‐swings (VS).[^9^, ^10^, ^11^] Alternative stimuli for carbon capture have been studied such as electrochemical pH swings,[^12^, ^13^, ^14^, ^15^] photochemical pH swings,^[16]^ polarity‐swings,[^17^, ^18^] and humidity‐swings.[^19^, ^20^, ^21^, ^22^] In the following discussions, we consider temperature‐swing DAC (TS‐DAC) in detail to identify the key challenges and how to address them.

### Thermodynamic Challenge of TS‐DAC

Thermodynamically, TS‐DAC is more energy demanding than TS‐post combustion capture (TS‐PCC):[^23^, ^24^, ^25^] in the capture stage, the sorbent must bind CO_2_ with a high capacity (such as >2 mol CO_2_/kg sorbent) at ambient temperature from air. Since *p*CO_2_ is lower in air (0.04 kPa) compared to in flue gas (13–15 kPa),^[9]^ air capture sorbents must possess a correspondingly higher binding energy in order to substantially load CO_2_ from air. In the regeneration stage, the sorbent must bind CO_2_ with a low capacity (such as <0.1 mol CO_2_/kg sorbent) at elevated temperatures ideally under an atmosphere of pure CO_2_ at 100 kPa. As air capture sorbents (e. g. KOH/K_2_CO_3_ cycle) are required to bind CO_2_ more strongly than PCC sorbents, a correspondingly higher temperature elevation is necessary to unload captured CO_2_ from the sorbent. In turn, this raises the thermal energy penalty necessary to drive a TS‐DAC process compared to a TS‐PCC process, which increases its cost of operation.

**Figure 1:**
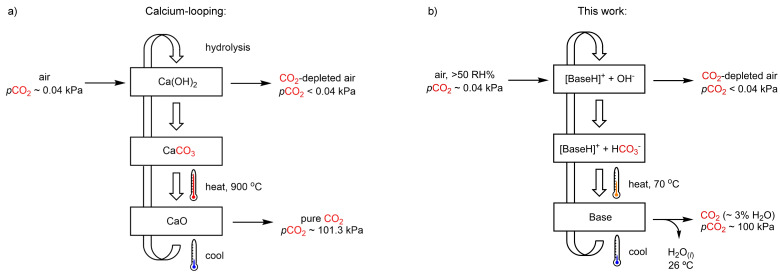
(a) Simplified schematic representation of calcium‐looping temperature‐swing direct air capture process (TSA‐DAC). (b) Schematic representation of this work.

Commercialized calcium‐looping DAC technology exhibits a high capture capacity of 18 mol/kg from air (CO_2_/CaO, Figure 1a),^[26]^ but at the expense of a high energy penalty during the regeneration step; the resulting CaCO_3_ must be exposed to temperatures as high as 900 °C via methane combustion to release pure CO_2_. In contrast, aqueous amine scrubbers release CO_2_ at much lower temperatures but at the expense of a low working capacity under DAC conditions; a solution of 30 wt% monoethanolamine (MEA) can achieve a maximum working capacity of ca. 0.4 mol/kg (CO_2_/solution) by releasing CO_2_ at 120 °C (*p*CO_2_=12 kPa).[^27^, ^28^]

Herein, an alternative TS‐DAC strategy is presented that uses water as a trigger to induce CO_2_ capture and desorption (Figure 1b). The system employs strongly basic peralkylated guanidines that capture up to one molar equivalent of CO_2_ from air only in the presence of sufficient amounts of moisture by forming guanidinium bicarbonates. Full desorption of CO_2_ occurs upon evaporation of water at 70 °C when water is condensed at <26 °C, affording an atmosphere of CO_2_ with a water vapor content of ca. 3 mol% at a total pressure of ~100 kPa.^[29]^ The use of water as a trigger permits the isolation of CO_2_ from air at a high partial pressure with a small temperature‐swing.

### Results and Discussions

#### Conceptualization

Aprotic nitrogen bases (e. g. peralkylated amidines/guanidines, tertiary amines) are innocent towards CO_2_ in the absence of water. However, the presence of water allows these bases to react with CO_2_ to form carbonate, CO_3_^2‐^, by effectively consuming two protons released from carbonic acid.[^30^, ^31^] The resulting carbonate anions are weakly basic (*p*K_aH_=10.3), and therefore do not further solubilize or capture CO_2_ from air efficiently.[^32^, ^33^, ^34^] As a result, strong bases (e. g. KOH or diazabicycloundecene, DBU) in aqueous solution capture theoretically up to 0.5 molar equivalents of CO_2_ from air affording the corresponding carbonate salts (K_2_CO_3_ or [DBUH]_2_[CO_3_]). From flue gas, (where *p*CO_2_ is 13–15 kPa), the same bases react with close to 1 molar equivalent of CO_2_ by forming bicarbonate salts.^[35]^

In contrast, when carbonate ions are not in aqueous solution and partially hydrated (e. g. in a hydrophobic environment) the basicity increases with several orders of magnitude.^[36]^ As a result, partially hydrated carbonate anions may react further with CO_2_ from air and form bicarbonate, HCO_3_^−^ despite a low *p*CO_2_.[^34^, ^36^, ^37^, ^38^] Based on these principles, it was postulated that strong aprotic organic bases could capture 1 molar equivalent of CO_2_ from air if the moisture content is sufficient.^[38]^ Thus, the addition of relative humidity (RH) as an additional component variable can be considered for a cyclic DAC process when studying the equilibrium properties of the system. Furthermore, removing water from the fully CO_2_‐loaded absorbents allows for complete recovery of all the captured CO_2_ without an induced vacuum or use of purge gasses.

#### Concept Verification

**Figure 2:**
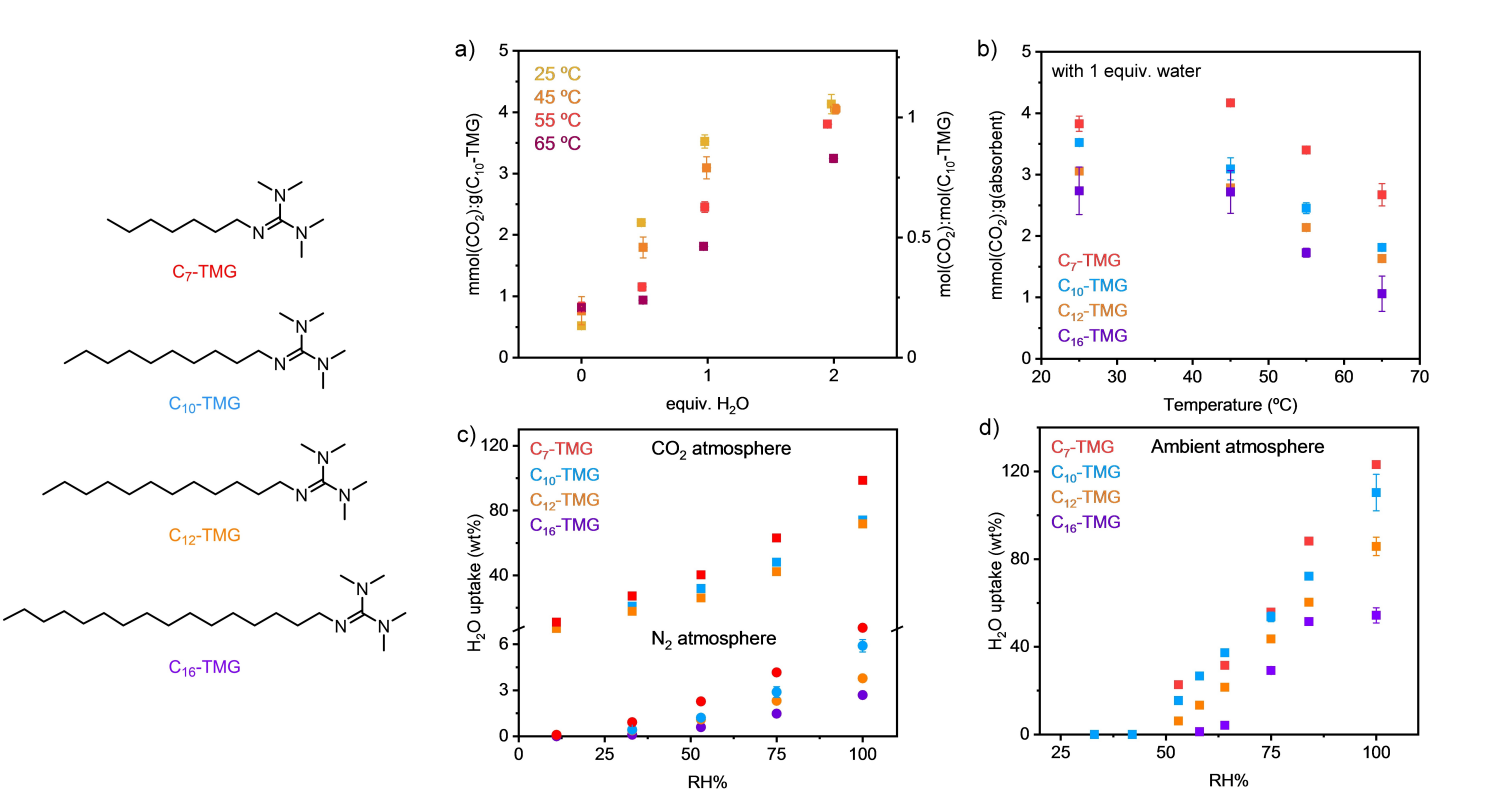
C_n_‐TMGs. (a) CO_2_ Capture capacity of C_10_‐TMG at different temperatures and water content under 1 atm CO_2_. (b) CO_2_ Capture capacity of C_n_‐TMGs with 1 equiv. H_2_O at different temperatures under 1 atm CO_2_. (c) Water uptake of C_n_‐TMGs under N_2_ atmosphere (circles) and CO_2_ atmosphere (squares) at 25 ^o^C. (d) Water uptake of C_n_‐TMGs under ambient air atmosphere at 25 ^o^C.

In this study, alkylated tetramethylguanidines (C_n_−TMG, C_n_=C_n_H_2n+1_, Figure 2) were chosen as model compounds to study the relationship between moisture content, temperature and CO_2_ capture capacity due to their strongly basic guanidine moiety suitable for DAC (*p*K_aH_~13.8 in water),^[39]^ negligible volatility, variable hydrophobicity and variable hygroscopicity (the latter two properties varies by the alkyl chain‐length, C_n_). The C_n_−TMGs were obtained as colorless, non‐viscous liquids by a one‐step alkylation of tetramethylguanidine (TMG) by alkylbromide (C_n_H_2n+1_Br) without sophisticated purification.^[40]^

The CO_2_ capture capacity of C_n_−TMGs were initially evaluated with different moisture content under an atmosphere of pure CO_2_ (ca. 1 atm=101.3 kPa). As expected, the CO_2_ capture capacity was near 0 in the absence of added moisture while it sharply increased upon introducing up to 2 equivalents of water (Figure S1). Further addition of water showed no notable improvement of CO_2_ loading, maintaining approximately 0.8 molar capacity in the presence of up to 60 equivalents of water. However, the CO_2_ capacity was highly sensitive to the moisture content between 0 and 2 equivalents of water. Thus, this water content region was further studied for C_10_−TMG at various temperatures (25–65 °C, Figure 2a). The temperature sensitivity of CO_2_ capacity was most prominent upon addition of 1 equivalent of water where a temperature‐swing of 40 °C resulted in a difference of 50 % in molar capacity (ca. 2 mmol/g). The same trend was observed for all C_n_−TMG analogues (Figure 2b). Analysis of ^13^C nuclear magnetic resonance spectroscopy of samples after CO_2_ absorption suggests that guanidinium (bi)carbonates were formed in D_2_O when CO_2_ reacts with C_n_−TMGs, which is in accordance with the gravimetric mass gain observed under a CO_2_ atmosphere. Independent infrared spectroscopic analysis of the CO₂ adducts further supported this claim (Figure S2). These initial experiments demonstrated a clear relationship between CO_2_ capacity of C_n_−TMGs and water content (humidity) and temperature.

To obtain further insight into the relationship between water content and CO_2_ capture capacity of C_n_−TMGs, the water uptake was studied under three different atmospheres (N_2_, air and CO_2_; Figures 2c–d) with variable relative humidity (RH) at a constant temperature of 25 °C. The water uptake capacity was highest for low molecular weight C_n_−TMGs (n=7>10>12>16) irrespective of the RH‐value and atmosphere. The water uptake was significantly lower under N_2_ as compared to air or CO_2_. The absence of CO_2_ reduces water uptake, similarly to how the absence of moisture reduces CO_2_ uptake by C_n_−TMGs. Interestingly, none of the C_n_−TMG analogues captured any detectable amounts of water below 40 % RH under ambient air (Figure 2d), indicating their low hygroscopicity. However, above 40 % RH, the water uptake increased linearly with the levels of RH, suggesting CO_2_ and water can be co‐absorbed above a critical RH‐value.

#### Direct Air Capture

**Figure 3:**
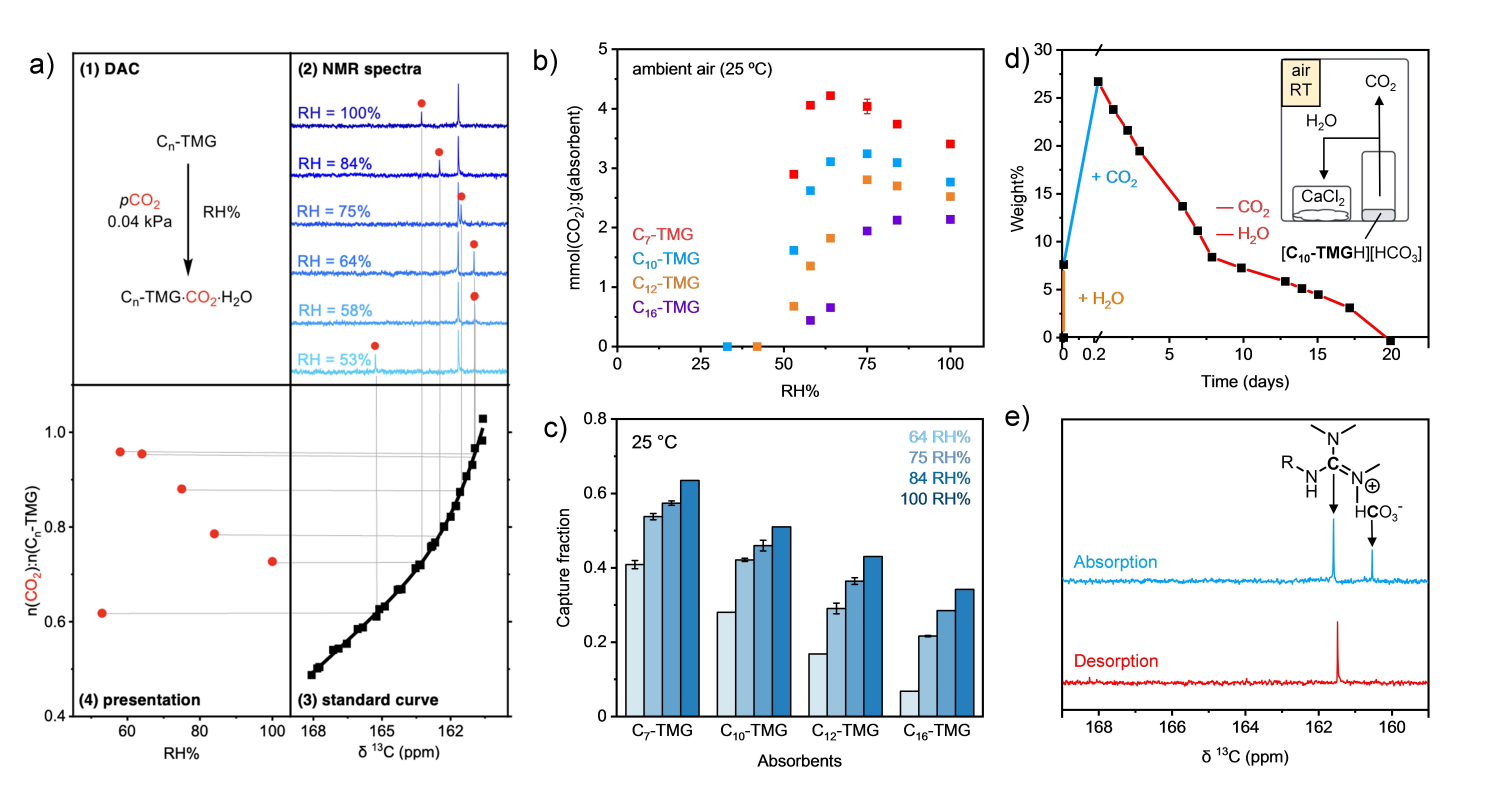
(a) ^13^C NMR (126 MHz) spectroscopic analysis of air capture CO_2_ loading of C_n_‐TMGs. (b) CO_2_ uptake by C_n_‐TMGs (0.050 g) upon passive exposure to a stream (3 mL min^−1^) of humidified air for 7 days at 25 °C. (c) Fraction of CO_2_ captured at various RH at approximately half of maximum capacity was reached. (d) Absorption and desorption of pre‐hydrated C_10_‐TMG. Addition of 1.1 equiv. H_2_O (orange line), absorption under 1 atm CO_2_ (blue line), desorption of CO_2_ and water from [C_10_‐TMGH][HCO_3_] with CaCl_2_ (red line) at ambient temperature. The mass of the system was periodically monitored and aliquots were withdrawn for NMR analysis. (e) Stacked ^13^C NMR spectra (126 MHz, D_2_O) of aliquots withdrawn from experiment after CO_2_ absorption (top) and after 10 days in closed container with CaCl_2_ (bottom). Complete disappearance of HCO_3_^−^/CO_3_^2−^ signal was observed.

Air capture capacity of C_n_−TMGs were evaluated by passively allowing samples to contact humidified air streams (3 mL ⋅ min^−1^) for 1 week and subsequently analyzing them by ^13^C NMR spectroscopy based on a standard curve (Figure 3a, see the SI for quantification method). The humid air stream was allowed to pass above the samples thereby contacting the surface of the absorbent (experimental setup in Figure S3).

The highest CO_2_ loading of each C_n_‐TMG analogue was observed at different RH‐values (Figure 3b, see Figure S4 for CO_2_ capacity of C_7_‐, C_10_‐ and C_12_‐TMG upon full equilibration in the range 50–100 % RH). As expected, the higher molecular weight C_n_‐TMGs required a higher RH‐value to reach their peak capture capacity; C_16_‐TMG showed its highest CO_2_ capacity above 80 % RH, C_7_‐ and C_10_‐TMG below 70 % RH, and C_12_‐TMG peaked between 70 %–80 % RH. This tendency is likely due to the increased hydrophobicity of the heavier C_n_‐TMGs, which in turn require higher humidity air to hydrate sufficiently to reach peak CO_2_ capture capacity.

The rate of CO_2_ captured from air (expressed as capture fraction, see SI for calculation details) was affected by a similar pattern for all C_n_‐TMGs; under our experimental conditions, the capture rate increased as the RH increased irrespective of equilibrium capacity (Figure 3c, Figures S5–9). It is important to note that the absolute capture rate can be increased drastically by simply increasing the flow rate and modifying the instrumentation to increase the contact areas. Our primary goal was to assess capture capacity and relative capture efficiency under a constant *p*CO_2_ (obtained via a low flow rate, see SI for more details on the instrumentation).

#### Low Temperature Regeneration of Absorbents

Since water addition and high humidity induces CO_2_ capture from the gas phase, the next step was to study if water removal and low humidity induces CO_2_ release from the sorbent phase (i. e. the reverse process). Initially, this was studied by placing a pre‐CO_2_ loaded sample of C_10_‐TMG in a dry sealed container containing CaCl_2_. The CO_2_‐rich C_10_‐TMG spontaneously and completely released CO_2_ at room temperature under these conditions demonstrating that water removal induces spontaneous desorption without raising the temperature (at room temperature, Figures 3d‐e). However, the head‐space gas evolved in this experiment was not pure CO_2_, which could not be obtained by simply removing water at ambient temperature. Therefore, the production of pure, concentrated (slightly moist) CO_2_ gas was demonstrated by optimizing the regeneration conditions under a CO_2_ atmosphere.

**Figure 4:**
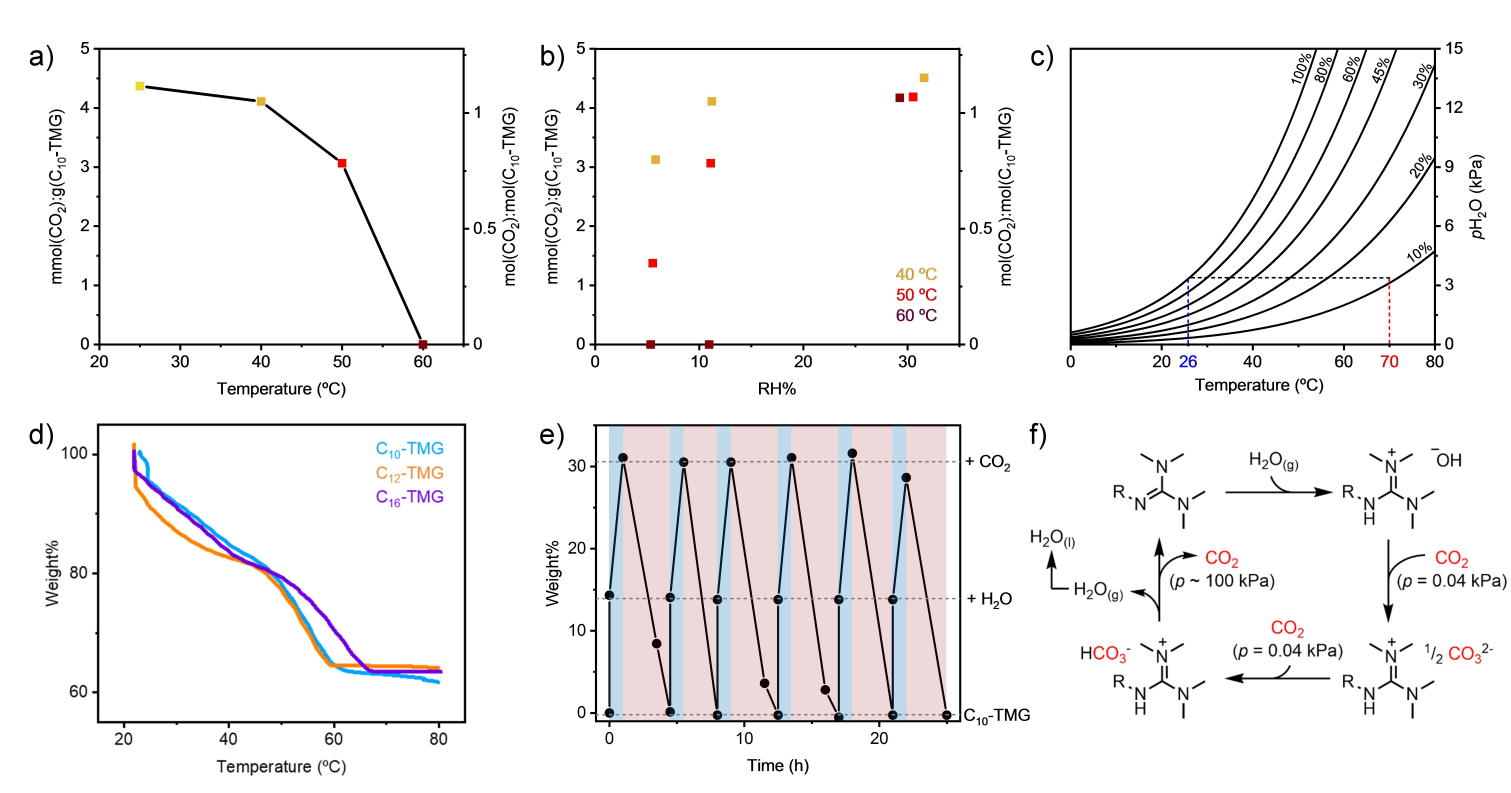
(a) CO_2_ Capacity of C_10_‐TMG under 1 atm CO_2_ at 11 % RH at various temperatures. (b) CO_2_ Capacity of C_10_‐TMG at various temperature and RH‐values. (c) Psychrometric chart showing the maximum temperature of a heat‐sink (26 °C, blue) that results in a relative humidity of <11 % at 70 °C. Lines depict RH %. (d) Thermogravimetric analysis (TGA) of C_10_‐, C_12_‐ and C_16_‐TMG after undergoing DAC at RH=75 %, 84 % and 100 %, respectively. Conditions: CO_2_ (100 %, 90 mL min^−1^), ramp 1 °C min^−1^. (e) Cyclic absorption/desorption by C_10_‐TMG under CO_2_. Absorption (blue): sample was moistened with 2 equiv. H_2_O, then placed under 1 atm CO_2_ at 25 °C for 1 hour. Desorption (red): sample was placed under 1 atm CO_2_ at 70 °C at a constant RH=11 %. (f) A proposed cycle for DAC cycle by the strong organic bases used in this work.

For C_10_‐TMG, isohume and isobaric CO_2_ loading under CO_2_ atmosphere (1 atm) was obtained at various temperatures (25–60 °C, 6 %–33 % RH depicted in Figures 4a‐b). Above 60 °C and below 11 % RH no CO_2_ absorption was detected after equilibration for 24 hours implying that desorption of pure CO_2_ is possible above 60 °C provided that the water content is below 11 % RH. However, when exposing pre‐CO_2_ loaded samples of C_n_‐TMGs to similar conditions, a temperature of 70 °C (11 % RH, 3 mol% H_2_O, dewpoint 26 °C) was necessary to induce full desorption of CO_2_ suggesting a path‐dependence (hysteresis) of CO_2_‐loading.^41^ The results suggest that full CO_2_ desorption can occur at 70 °C, yielding undiluted CO_2_ containing 3 mol% H_2_O, provided that the process is equipped with a heat sink below the dew‐point temperature for water condensation (Figure 4c).^29^

Thermogravimetric analysis (TGA) of CO_2_/H_2_O adducts – hydrated guanidinium bicarbonates, independently prepared from DAC experiments – showed water/CO_2_ co‐desorption with a theoretical mass loss of CO_2_ and water under a >99.9 % CO_2_ stream (1 atm) at a decomposition temperature of 55–65 °C (Figure 4d). This result suggests that a mere 45 °C temperature swing is sufficient to achieve a >90 % working capacity of C_n_‐TMG analogues (up to 4.5 mol CO_2_/kg absorbent or 21 wt % gravimetric capacity).

#### Cyclic Experiments and Aqueous Stability

A small scale cyclic experiment under CO_2_ atmosphere using C_10_‐TMG showed that addition of water (2 equiv.) at 25 °C favors CO_2_ capture, whereas drying the atmosphere at 70 °C favors complete release of CO_2_ without changing the atmosphere (Figure 4e). It is noteworthy here that the outcome of this experiment manifests that pure CO₂ generation is possible under the control of relative humidity and temperature of the system.

The stability of aqueous C_10_‐TMG was evaluated to show the application potential: under acidic and basic pHs; C_10_‐TMG was found to be stable for months at low *p*Hs (<1), whereas it decomposes before two months at elevated pH presumably via hydrolysis to form thermodynamically stable urea.

#### Proposed Cycle

We postulate a DAC cycle with C_n_‐TMG (R = alkyl, Figure 4f). Initially, the C_n_‐TMGs are hydrated by moisture from the air. Once sufficiently hydrated, CO_2_ is captured by hydroxide anions as CO_3_^2‐^, which generates a liquid and ionic substance.[^42^, ^43^] The hydrated CO_3_^−2^ further reacts with atmospheric CO_2_ generating HCO_3_^−^. Depending on the relative humidity, the hydration and basicity of CO_3_^2‐^ changes, impacting the CO_2_ capture capacity of the guanidine base.[^34^, ^36^, ^37^, ^38^] Once fully loaded, concentrated (moist) CO_2_ is released by evaporating water at elevated temperature (ca. 70 °C) and condensing water until the water vapor content of the atmosphere is below 3 mol%, whereby all captured CO_2_ and water is released from the C_n_‐TMG absorbent.

The formation of guanidinium hydroxides was qualitatively supported by conductivity measurements of wetted C_10_‐TMG, wherein the conductivity increased exponentially beyond addition of 0.2 molar equivalents of water (Figure S10). This increase of conductivity is expected when ionic species are formed and highlights the impact of water as a trigger to induce Arrhenius basicity of non‐nucleophilic hydrophobic organic bases.

## Conclusions

We successfully demonstrated the use of strong organic bases, C_n_‐TMGs, for direct air capture enabled by ambient relative humidity and a small temperature swing. Equilibration of C_n_‐TMGs under moist air shows that CO_2_ capture capacity changes drastically depending on the RH‐value of the atmosphere and hydrophobicity of the absorbent. High working capacity and gravimetric capacity were achieved with lipophilic strong organic bases, which exhibit low vapor pressure and volatility. Further evaluation and design of a continuous system is underway in a larger scale DAC process with optimized air‐contactor for improved mass transport of CO₂.

## Conflict of Interests

The authors declare competing interests with a preliminary patent application (EP24206054.9)

## Supporting information

As a service to our authors and readers, this journal provides supporting information supplied by the authors. Such materials are peer reviewed and may be re‐organized for online delivery, but are not copy‐edited or typeset. Technical support issues arising from supporting information (other than missing files) should be addressed to the authors.

Supporting Information
