# Supplementary material for: Water‐Triggered Direct Air Capture by Strong Organic Bases
Source: ChemSusChem. 2025 Feb 18;18(11):e202402685. doi: 10.1002/cssc.202402685 (PMC12131680; doi:10.1002/cssc.202402685)
Supplement: Supplementary file 1 — Supporting Information [file CSSC-18-e202402685-s001.pdf]

ChemSusChem

Supporting Information

Water-Triggered Direct Air Capture by Strong Organic Bases

Anders Grundtvig Utzon and Ji-Woong Lee*

Water-Triggered Direct Air Capture by Strong Organic Bases

Anders Grundtvig Utzon,[1,3] Ji-Woong Lee[1,2,3]*

[1]Department of Chemistry, University of Copenhagen, Universitetsparken 5, Copenhagen Ø, 2100, Denmark

[2]Nanoscience Center, University of Copenhagen, Universitetsparken 5, Copenhagen Ø, 2100, Denmark

[3]Novo Nordisk Foundation CO_2 Research Center, Gustav Wieds Vej 10, Aarhus 8000, Denmark

E-mail: jiwoong.lee@chem.ku.dk

Table of Contents

**1**	**Materials and Methods**	**…………..**	**2**
**2**	**Synthesis procedures**	**…………..**	**6**
**3**	**Carbon capture capacity of absorbents with variable water content and infrared spectra**	**…………..**	**8**
**4**	**Graphical representation of experimental setup for Direct Air Capture**	**…………..**	**9**
**5**	**Full carbon capture capacity of absorbents from humid air**	**…………..**	**10**
**6**	**Kinetic data from Direct Air Capture experiments**	**…………..**	**11**
**7**	**Conductivity of C_{10}-TMG**	**…………..**	**14**
**8**	**Extended discussion of quantification method**	**…………..**	**15**
**9**	**Standard curve for CO_2 loading determination by ^{13}C NMR Spectroscopy**	**…………..**	**18**
**10**	**Graphical representation of experimental setup for humidity regulated capture experiments**	**…………..**	**20**
**11**	**NMR Spectra of absorbents**	**…………..**	**21**
**12**	**Infrared Spectra of absorbents**	**…………..**	**25**
**13**	**^{13}C NMR spectra of CO_2-loaded absorbents**	**…………..**	**27**
**14**	**Raw kinetic data from Direct Air Capture experiments**	**…………..**	**30**

1. Materials and Methods

^{1}H NMR and ^{13}C NMR spectra were recorded at 500 MHz and 126 MHz respectively using $CDCl_3$, D_2O and MeCN-d_3 as solvents on a Bruker Ultrashield Plus 500 spectrometer. All chemical shifts (δ) are reported in ppm using the solvent residual peak as a reference (or ethylene glycol 62.50 ppm for ^{13}C NMR in D_2O where guanidinium carbons resonate at 161.63, 161.59, 161.55 and 161.54 ppm for C_7-, C_{10}-, C_{12}- and C_{16}-TMG respectively) and all coupling constants (J) are expressed in Hertz (Hz). The following abbreviations are used for multiplicity for NMR resonances: s = singlet, d = doublet, t = triplet, q = quartet, and m = multiplet. Fourier transform infrared (FT-IR) spectra were collected on a Bruker Alpha-P FT-IR spectrometer equipped with an attenuated total reflectance (ATR) module. The thermogravimetric analysis (TGA) was performed using a Discovery TGA from TA instruments (New Castle, DE, USA). The samples were heated in a platinum TGA pan. LC-MS analyses were carried out by connecting the above mentioned HPLC apparatus to a Bruker MicrOTOF-QII system equipped with an ESI source with nebulizer gas at 1.2 bar, dry gas at 10 L/min, dry temperature at 200 °C, capillary at 4500 V and end plate offset at -500 V. The ion transfer was conducted with funnel 1 and funnel RF's at 200.0 Vpp and hexapole RF at 100.0 Vpp while the quadrupole ion energy was set at 5.0 eV with a low mass cut-off at 100.00 m/z. In the collision cell, collision energy was set at 8.0 eV, collision RF at 100.0 Vpp, and a transfer time of 80.0 µs and pre-pulse storage of 1.0 µs were used. High-resolution mass spectrometry was performed on a Bruker SolariX XR 7 T ESI/MALDIFT-ICR-MS instrument. Conductivity measurements were performed using HI-2003 Edge® Conductivity Meter. Both portable meters were purchased from Hanna instrument, Denmark including the standard solution for calibration of conductivity probe (HI-7030 to 12880 µS cm^{-1} at 25°C). Solvents, reagents, and chemicals were purchased from commercial vendors and used without further purification. CO_2 (99.999%) and compressed air (ca. 420 ppm CO_2) was purchased from Air Liquide and used as received. N_2 was supplied in-house. Absorbents were stored tightly capped laboratory bottles at 5 ^{o}C. Laboratory bottles containing absorbents were removed from refrigerator approximately 30 minutes prior to use or until they reached ambient temperature.

Procedure for CO_2 uptake under CO_2 atmosphere with various water amounts added

To a 20 mL vial, 0.500 g absorbent and water (0-2 equiv.) was added, as well as a magnetic stir bar. The vial was sealed with a screw-cap containing a septum and the total mass was recorded. The absorbent and water mixtures were briefly stirred to homogenize, where after the system was purged with CO_2 supplied by a freshly prepared double-wrapped balloon for 30 seconds at ambient temperature. After purging, the vial was tightly sealed and placed in a pre-heated aluminum block at the desired temperature (25, 45, 55 or 65 ^{o}C) and left to stir under CO_2 atmosphere. The vials were insulated by cotton and aluminum foil to maintain a constant temperature. After stirring for 20 hours, the vial was removed from the heating block.

The balloon was immediately thereafter removed and the head-space atmosphere was quickly exchanged with air by opening the vial for ca. 5 seconds to avoid any further CO_2 uptake or water evaporation upon cooling. The mass of the vial was recorded. The experiments were reproduced in triplicates.

Procedure for Direct Air Capture experiments

Air was supplied by a pressurized air tank containing ~420 ppm CO_2. For DAC experiments, the flow-rate was adjusted to 3 mL min^{-1} at 25 ºC (2.748 sccm) by a flow-controller (N_2 as reference gas). Air was passed into a 250 mL gas wash bottle containing approximately 45 mL of a saturated salt solution to regulate the relative humidity. The following saturated salt solutions were used to regulate the relative humidity: $MgCl_2$ (33 RH%), $ZnCl_2$ (42 RH%), $Mg(NO_3)_2$ (53 RH%), NaBr (57 RH%), NH_4NO_3 (64 RH%), NaCl (75 RH%), KCl (84 RH%) and deionized water (100 RH%).[44] Before an experiment, the system was allowed to equilibrate by purging air through the system for up to 24 hours. The entire system was kept at a constant temperature of 25 ºC by a temperature-controlled water-bath. The humidified air was passed over an 8 mL vial containing 0.050 g of absorbent placed at the tip of another gas wash bottle. The effluent air was passed through a small bubbler containing 2-4 mL water and 1 drop of H_2SO_4. The mass of the vial was recorded before and after the experiments while sealed to prevent mass loss/gain to the ambient atmosphere. For NMR spectroscopic analysis, the absorbent was dissolved in D_2O to provide a molal concentration of roughly 0.090 mol kg^{-1}. Graphical representation of the experimental setup is shown in Fig. S3.

For kinetics experiments, each data point corresponds to one experiment.

Procedure for quantification of CO_2 and water uptake from direct air capture experiments

The total mass of the vial was recorded before and after each experiment. The entire contents of the vial was then dissolved in D_2O to give a molal concentration of 0.090 mol(absorbent) kg^{-1}. The CO_2 uptake was calculated by ^{13}C NMR spectroscopic chemical shift of HCO_3^-/CO_3^{2-} using a standard curve (Fig. S12). The water uptake was quantified by subtracting the CO_2 uptake from the total mass gain. An extensive description of the CO_2 uptake quantification method and an in-depth discussion on choice of method is provided in sections 8-9 in the Supplementary Information.

Procedure for water uptake studies under N_2 and CO_2 atmosphere at different relative humidity

To a 4 mL vial, 0.100 g absorbent was added, as well as a magnetic stir bar. The mass of the system was recorded. The vial was placed in a preheated 40 mL vial at 25 ºC containing a wetted salt (or water) to regulate the relative humidity. The following wetted salts were used to regulate the relative humidity: LiCl (11 RH%), $MgCl_2$ (33 RH%), $Mg(NO_3)_2$ (53 RH%), NaCl (75 RH%), KCl (84 RH%) and deionized water (100 RH%)[44] The system was purged with CO_2 or N_2 supplied by a freshly prepared double-wrapped

balloon. The vials were insulated by cotton and aluminum foil to maintain a constant temperature of 25 ºC. No further water-uptake occurred after 24 hours under N_2, while the system was allowed to equilibrate for 7 days under CO_2 atmosphere. The water uptake was quantified by recording the mass gain. For samples under CO_2 atmosphere, the CO_2 uptake (quantified by NMR) was subtracted from the mass gain. Graphical representation of the experimental setup is shown in Fig. S13.

Procedure for determination of CO_2 capacity under constant relative humidity and variable temperature

To a 4 mL vial, 0.200 g C_{10}-TMG was added, as well as a magnetic stir bar. The mass of the system was recorded. The vial was placed in a preheated 20 mL vial at 80 ºC containing a wetted salt to regulate the relative humidity. The following wetted salts were used to regulate the relative humidity: LiBr (5.3-5.8 RH%), LiCl (10.5-11.3 RH%), $MgCl_2$ (26.1-32.8 RH%).[44] The system was insulated by cotton and aluminum foil to maintain a constant temperature. The system was purged with CO_2 for 90 seconds supplied by a freshly prepared double-wrapped balloon and allowed to equilibrate for 24 hours. After 24 hours, an aliquot was withdrawn and analyzed by NMR spectroscopy to quantify CO_2 capacity. The mass of the system was recorded. Using the same sample, the entire procedure was repeated again at 70 ºC, 60 ºC, 50 ºC and finally 40 ºC by lowering the temperature of the aluminum block by 10 ºC between each experiment. Graphical representation of the experimental setup is shown in Fig. S13.

Desorption experiments

Absorption. To a 4 mL vial, 0.100 g absorbent (C_7-, C_{10}- and C_{12}-TMG) and water (2 equiv.) was added, as well as a magnetic stir bar. The vial was sealed with a screw-cap containing a septum and the total mass was recorded. The absorbent and water mixtures were briefly stirred to homogenize, where after the system was purged with CO_2 supplied by a freshly prepared double-wrapped balloon for 5 seconds at ambient temperature. After purging, the vial was tightly sealed and placed in a pre-heated aluminum block at 25 ºC and left to stir under CO_2 atmosphere. After stirring for 2 hours the vial was removed from the heated aluminum block. The balloon was removed and the head-space atmosphere was quickly exchanged with air by opening the vial for ca. 5 seconds. The mass of the vial was recorded corresponding to the CO_2 absorption (mol(CO_2):mol(C_n-TMG) was 1.15, 1.18 and 1.01 for n = 7, 10, and 12 respectively). *Desorption.* Hereafter the 4 mL vials containing CO_2-rich C_n-TMG were placed in a 20 mL vial containing wetted LiCl at 70 ºC and rapidly supplied with a CO_2 atmosphere by purging for 90 seconds. Desorption of CO_2 and water was complete after 3 hours (C_{10}- and C_{12}-TMG) or 7 hours (C_7-TMG). *It is worth noting that the physical appearance of the samples resemble that of the CO_2-free absorbent (non-viscous liquid) after stirring at 70 ºC and 11 RH%. Additionally, the samples of C_{10} and C_{12}-TMG were poorly soluble in D_2O, which suggests complete CO_2 desorption. NMR spectra (126 MHz, 1024 scans) in MeCN-d_3 (C_{10}- and*

C_{12}-TMG) or D_2O (C_7-TMG) revealed no detectable CO_2 species. Graphical representation of the experimental setup used for the desorption step is shown in Fig. S13.

Simulated cycling experiments

Absorption. To a 4 mL vial, 0.0747 g C_{10}-TMG and water (0.0107 g, 2 equiv.) was added, as well as a magnetic stir bar. The vial was sealed with a screw-cap containing a septum and the total mass was recorded. The absorbent and water mixtures were briefly stirred to homogenize, where after the system was purged with CO_2 supplied by a freshly prepared double-wrapped balloon for 5-10 seconds at ambient temperature. After purging, the vial was tightly sealed and placed in a pre-heated aluminum block at 25 ºC and left to stir under CO_2 atmosphere. After stirring for 1 hours the vial was removed from the heated aluminum block. The balloon was removed and the head-space atmosphere was quickly exchanged with air by opening the vial for ca. 5 seconds. The mass of the vial was recorded. *Desorption.* Hereafter the system was placed in a 20 mL vial containing wetted LiCl at 70 ºC and rapidly supplied with a CO_2 atmosphere by purging for 90 seconds. After 2-3 hours, the vial was removed and the mass was recorded.

The procedure was repeated on the same sample. Graphical representation of the experimental setup used for the desorption step is shown in Fig. S13.

Procedure for thermogravimetric analysis under CO_2 atmosphere

The thermogravimetric analysis (TGA) was performed under a constant flow of 90 mL min^{-1} CO_2. The samples were heated in a platinum TGA pan from room temperature to 80 °C at a heating rate of 1 °C min^{-1}.

Procedure for stability studies

Stability under acidic pH: to 0.75 g 3.8 M H_2SO_4 in D_2O was added 65.4 mg C_{10}-TMG.

Stability under basic pH: to 0.63 g 2.5 M KOH in D_2O was added 52.4 mg C_{10}-TMG.

The samples were analyzed by NMR spectroscopy at regular intervals.

2. Synthesis procedures

General procedure for synthesis of alkylated tetramethylguanidines

1,1,3,3-tetramethylguandidine (TMG) was alkylated according to a slightly modified literature procedure.[40] To a flame-dried 500 mL round bottomed flask equipped with a reflux condenser and an addition funnel, ~60 mL TMG (0.478 mol, 2.3 equiv.) was dissolved in 45 mL xylenes (or toluene) and placed in a preheated 125 ºC aluminum block under nitrogen atmosphere. While stirring, alkylbromide (0.211 mol, 1 equiv.) was added dropwise over 2-4 hours and left to stir overnight. The solids were removed by gravity filtration. The solvent (o-xylene or toluene) was removed under reduced pressure and product was isolated via simple distillation. For the heavier boiling analogues, a white solid precipitates from the distillate upon cooling which was removed by gravity filtration. *No difference in yield was observed when alternating between o-xylene and toluene as solvent.*

2-heptyl-1,1,3,3-tetramethylguanidine (C_7-TMG):

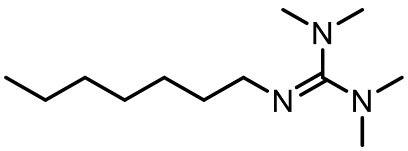

Compound was synthesized according to general synthesis procedure using TMG (65 mL, 0.518 mol, 2.1 equiv.) and 1-bromoheptane (38 mL, 0.242 mol, 1 equiv.). The product was isolated as a colorless, non-viscous liquid. Yield: 23.7025 g (46%). Boiling point: 74-79 ºC (0.83 mm Hg). ^{1}H NMR (500 MHz, $CDCl_3$) δ 3.07 (t, J = 7.00 Hz, 2H), 2.71 (s, 6H), 2.62 (s, 6H), 1.49 (p, J = 7.03 Hz, 2H), 1.32-1.19 (m, 8H), 0.86 (t, J = 7.0 Hz, 3H). ^{13}C NMR (126 MHz, $CDCl_3$) δ 160.01, 49.78, 39.76, 38.94, 32.96, 32.10, 29.37, 27.60, 22.79, 14.23. FT-IR (neat): 1360, 1621, 2854, 2923 cm^{-1}. Elemental analysis. Anal. Calcd $C_{12}H_{27}N_3$: C, 67.55; H, 12.76; N 19.69. Found: C, 64.66; H, 12.61; N, 18.90. HRMS [M+H$^+$]: Calcd 214.22832, found 214.22786.

2-decyl-1,1,3,3-tetramethylguanidine (C_{10}-TMG):

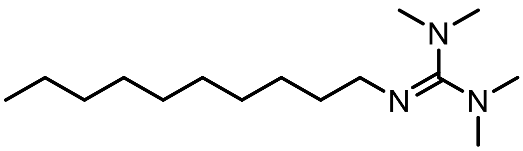

Compound was synthesized according to general synthesis procedure using TMG (60 mL, 0.478 mol, 2.2 equiv.) and 1-bromodecane (45 mL, 0.218 mol, 1 equiv.). The product was isolated as a colorless, non-viscous liquid. Yield: 27.4207 g (49%). Boiling point: 106-110 ºC (0.50 mm Hg). ^{1}H NMR (500 MHz, $CDCl_3$) δ 3.08 (t, J = 6.98 Hz, 2H), 2.72 (s, 6H), 2.63 (s, 6H), 1.49 (p, J = 7.00 Hz, 2H), 1.32-1.19 (m, 14H),

0.86 (t, *J* = 6.92 Hz, 3H). ^{13}C NMR (126 MHz, $CDCl_3$), δ 160.03, 49.77, 39.78, 38.96, 32.95, 32.06, 29.87, 29.77, 29.71, 29.49, 27.65, 22.82, 14.24. FT-IR (neat): ν =1360, 1621, 2852, 2922 cm^{-1}. Elemental analysis. Anal. Calcd $C_{15}H_{33}N_3$: C, 70.53; N 16.45. Found: C, 72.04; N, 16.64. HRMS [$M+H^+$]: Calcd 256.27527, found 256.27476.

2-dodecyl-1,1,3,3-tetramethylguanidine (C_{12}-TMG):

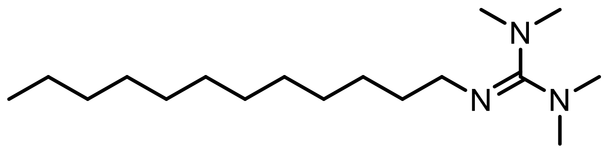

Compound was synthesized according to general synthesis procedure using TMG (60 mL, 0.478 mol, 2.3 equiv.) and 1-bromododecane (49 mL, 0.204 mol, 1 equiv.). The product was isolated as a colorless, non-viscous liquid. Yield: 29.4928 g (51%). Boiling point: 125-136 ºC (0.98 mm Hg). ^{1}H NMR (500 MHz, $CDCl_3$) δ 3.08 (t, *J* = 6.99 Hz, 2H), 2.73 (s, 6H), 2.64 (s, 6H), 1.50 (p, *J* = 7.1 Hz, 2H), 1.32-1.18 (m, 18H), 0.87 (t, *J* = 6.91 Hz, 3H). ^{13}C NMR (126 MHz, $CDCl_3$) δ 160.05, 49.78, 39.79, 38.98, 32.96, 32.07, 29.89, 29.85, 29.82, 29.81, 29.72, 29.50, 27.66, 22.83, 14.25. FT-IR (neat): 1360, 1622, 2852, 2921 cm^{-1}. Elemental analysis. Anal. Calcd $C_{17}H_{37}N_3$: C, 72.02; H, 13.16; N 14.82. Found: C, 71.21; H, 13.31; N, 14.34. HRMS [$M+H^+$]: Calcd 256.27527, found 256.27476.

2-hexadecyl-1,1,3,3-tetramethylguanidine (C_{16}-TMG):

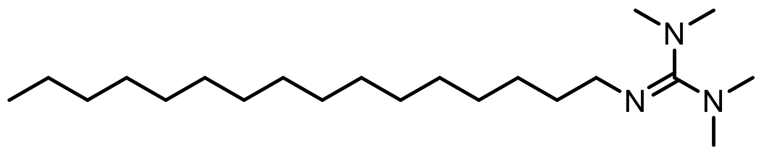

Compound was synthesized according to general synthesis procedure using TMG (60 mL, 0.478 mol, 2.2 equiv.) and 1-bromohexadecane (66 mL, 0.216 mol, 1 equiv.). The product was isolated as a colorless, non-viscous liquid. Yield: 35.6556 g, 49%. Boiling point: 170-180 ºC (1.13 mm Hg). ^{1}H NMR (500 MHz, $CDCl_3$) δ 3.08 (t, *J* = 7.01 Hz, 2H), 2.72 (s, 6H), 2.63 (s, 6H), 1.49 (p, *J* = 7.15 Hz, 2H), 1.31-1.18 (m, 26H), 0.87 (t, *J* = 6.99 Hz, 3H). ^{13}C NMR (126 MHz, $CDCl_3$) δ 160.01, 49.81, 39.78, 38.96, 32.98, 32.06, 29.89, 29.84 (multiple overlapping peaks), 29.82, 29.80, 29.72, 29.50, 27.66, 22.82, 14.24. FT-IR (neat): 1360, 1623, 2851, 2921 cm^{-1}. Elemental analysis. Anal. Calcd $C_{21}H_{45}N_3$: C, 74.27; H, 13.36; N 12.37. Found: C, 72.64; H, 13.16; N, 11.53.

3. Carbon capture capacity of absorbents with variable water content and infrared spectra

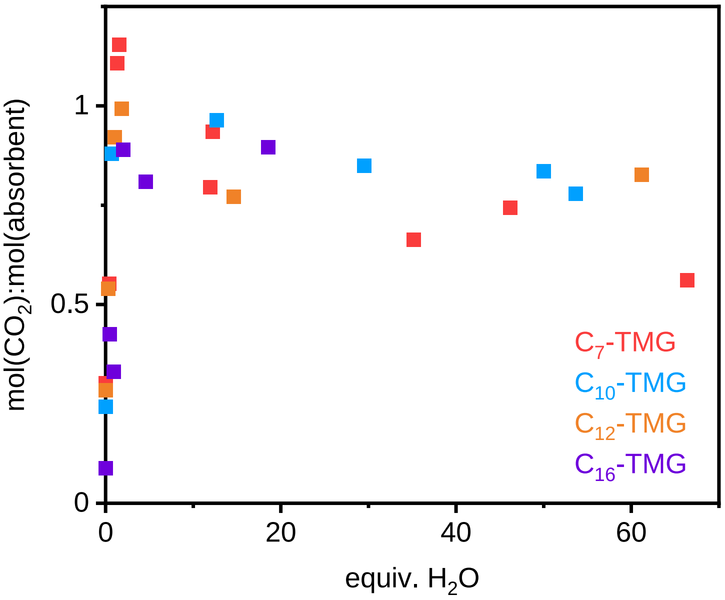

Fig. S1. CO_2 capacity of C_n-TMG absorbents (0.200 g scale) at ambient temperature under an atmosphere of CO_2.

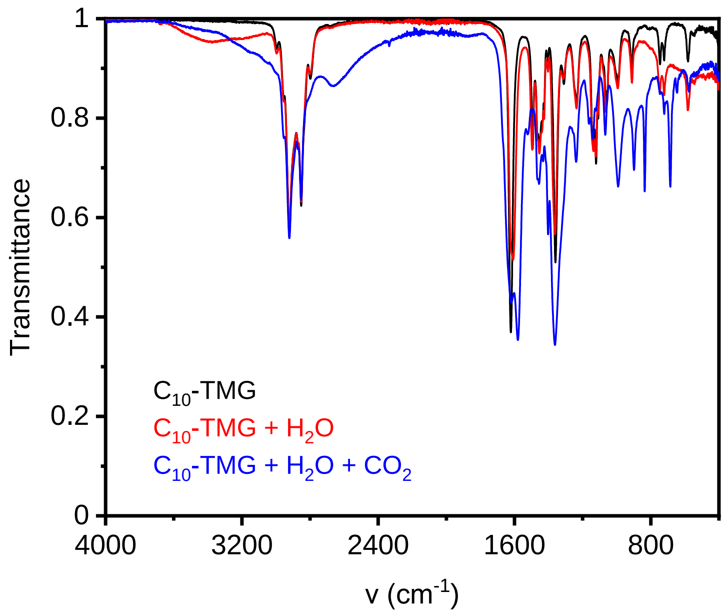

Fig. S2. IR Spectra (neat) of C_{10}-TMG (black); C_{10}-TMG upon stirring for 5 hours with 1 equiv. of water (red); C_{10}-TMG upon stirring for 5 hours with 1 equiv. of water under CO_2 atmosphere (blue).

4. Graphical representation of experimental setup for Direct Air Capture

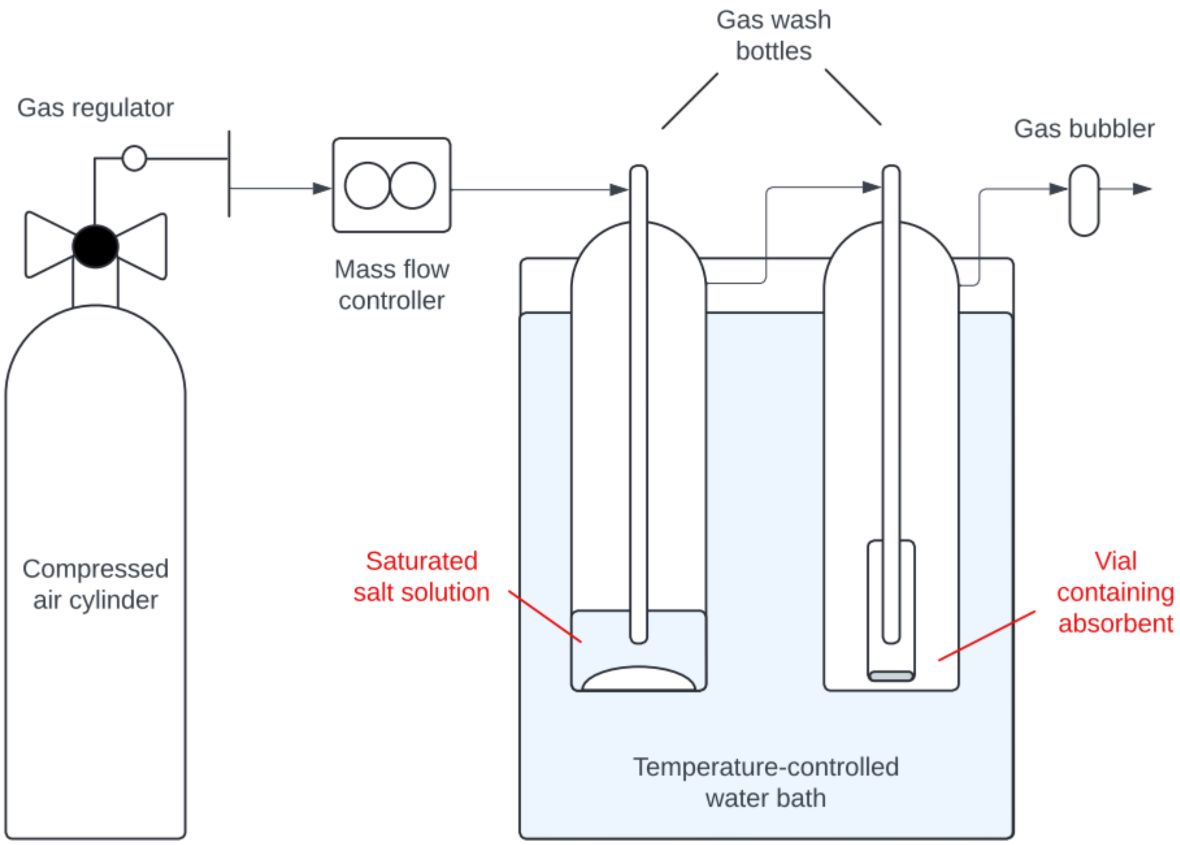

Fig. S3. Graphical representation of experimental setup for direct air capture experiments.

5. Full carbon capture capacity of absorbents from humid air

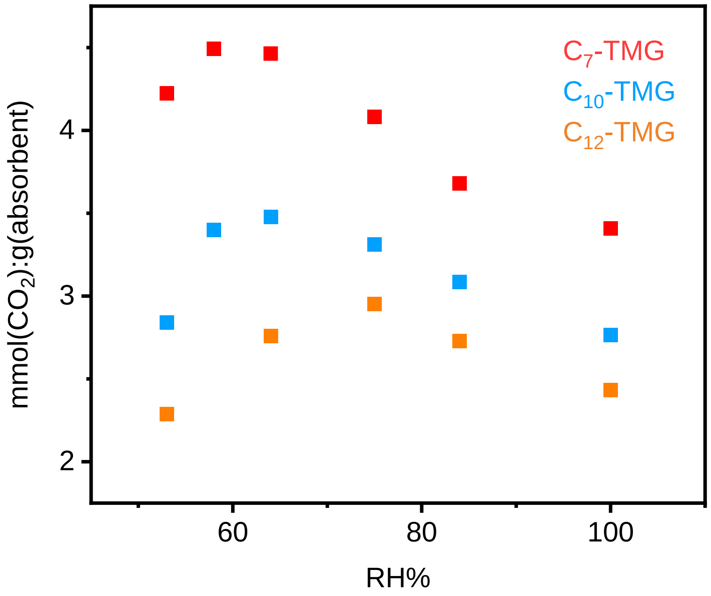

Fig. S4. CO_2 capacity of **C_7-**, **C_{10}-** and **C_{12}-TMG** from air at various relative humidity upon reaching full equilibrium. Due to extensive equilibration times, **C_{16}-TMG** is omitted.

6. Kinetic data from Direct Air Capture experiments

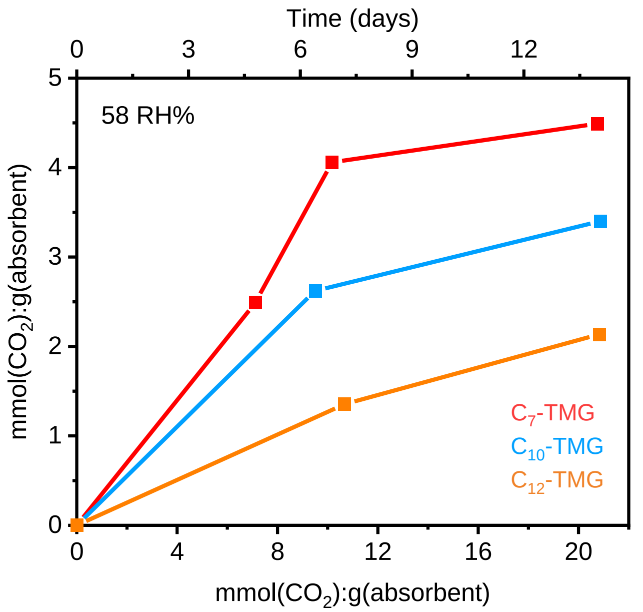

Fig. S5. Abscissa: amount of CO_2 (mmol) passed over absorbent (pr. gram) from air (RH = 58%, 25 ºC) at a flow rate of 3 mL min^{-1} (bottom), and the corresponding time stamp (top). Ordinate: CO_2 loading (mmol/g) of absorbent.

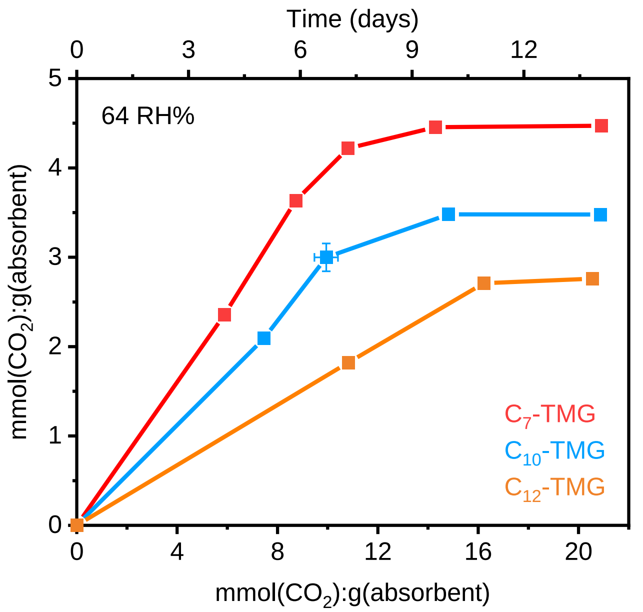

Fig. S6. Abscissa: amount of CO_2 (mmol) passed over absorbent (pr. gram) from air (RH = 64%, 25 ºC) at a flow rate of 3 mL min^{-1} (bottom), and the corresponding time stamp (top). Ordinate: CO_2 loading (mmol/g) of absorbent.

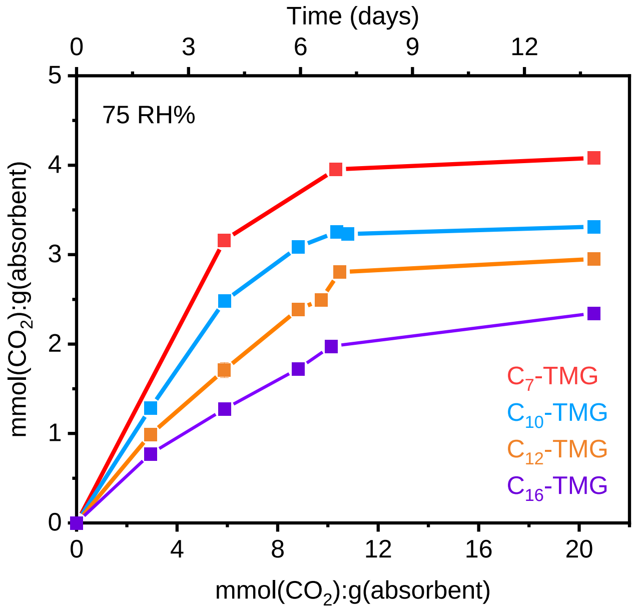

Fig. S7. Abscissa: amount of CO_2 (mmol) passed over absorbent (pr. gram) from air (RH = 75%, 25 ºC) at a flow rate of 3 mL min^{-1} (bottom), and the corresponding time stamp (top). Ordinate: CO_2 loading (mmol/g) of absorbent.

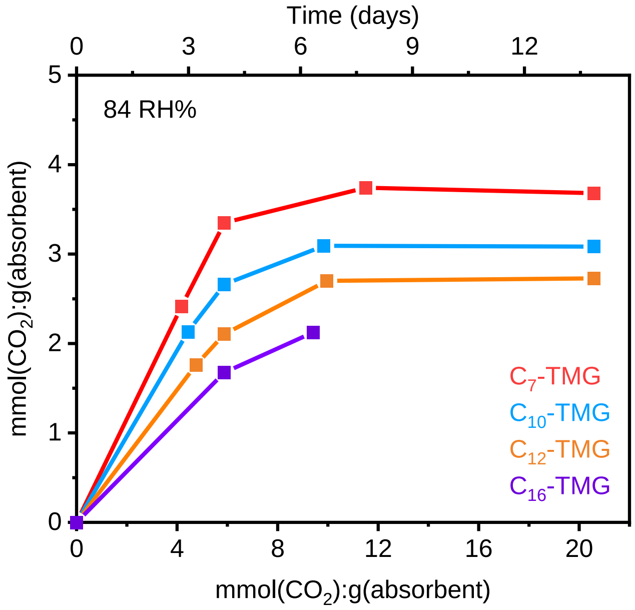

Fig. S8. Abscissa: amount of CO_2 (mmol) passed over absorbent (pr. gram) from air (RH = 84%, 25 ºC) at a flow rate of 3 mL min^{-1} (bottom), and the corresponding time stamp (top).. Ordinate: CO_2 loading (mmol/g) of absorbent.

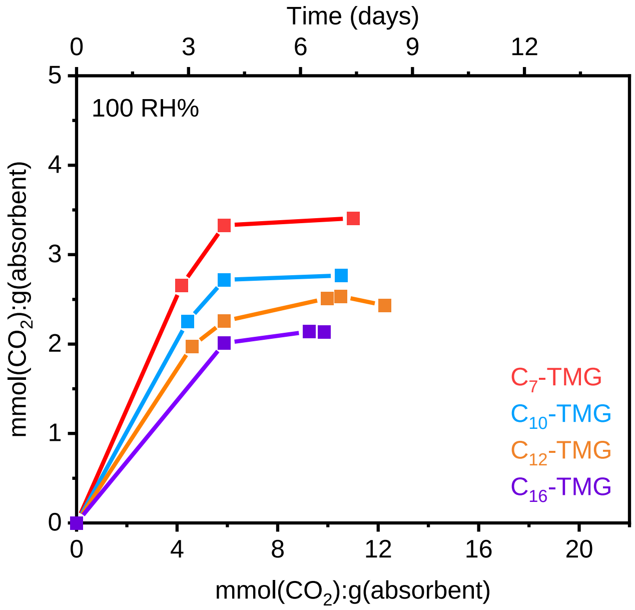

Fig. S9. Abscissa: amount of CO_2 (mmol) passed over absorbent (pr. gram) from air (RH = 100%, 25 ºC) at a flow rate of 3 mL min^{-1} (bottom), and the corresponding time stamp (top). Ordinate: CO_2 loading (mmol/g) of absorbent.

Capture fraction calculation

The air capture rate was roughly constant until near the saturation point where the rate diminished abruptly. The rate of air capture during this initial stage was quantified as a ratio between the amount of CO_2 captured $n(CO_2)_{cap}$ and the amount of CO_2 passed over the sample $n(CO_2)_{tot}$ at roughly half capacity denoted "capture fraction":

$$capture\ fraction = \frac{n(CO_2)_{cap}}{n(CO_2)_{total}} = \alpha * \text{n}(\text{C}_\text{n}\text{-TMG}) * \frac{R*T}{p*Q*t*x(CO_2)} \text{ (eq.1)}$$

Where α is the molar CO_2 loading obtained by NMR quantification, $n(C_n\text{-TMG})$ is the moles of C_n-TMG, p = 1 atm is the gas pressure, R = 0.0821 L atm mol^{-1} K^{-1} is the gas constant, T = 298 K is the absolute temperature , Q = 3 mL min^{-1} is the flow rate, t is the time, $x(CO_2) = 4.2*10^{-4}$ is the mole fraction of CO_2.

7. Conductivity of C_{10}-TMG

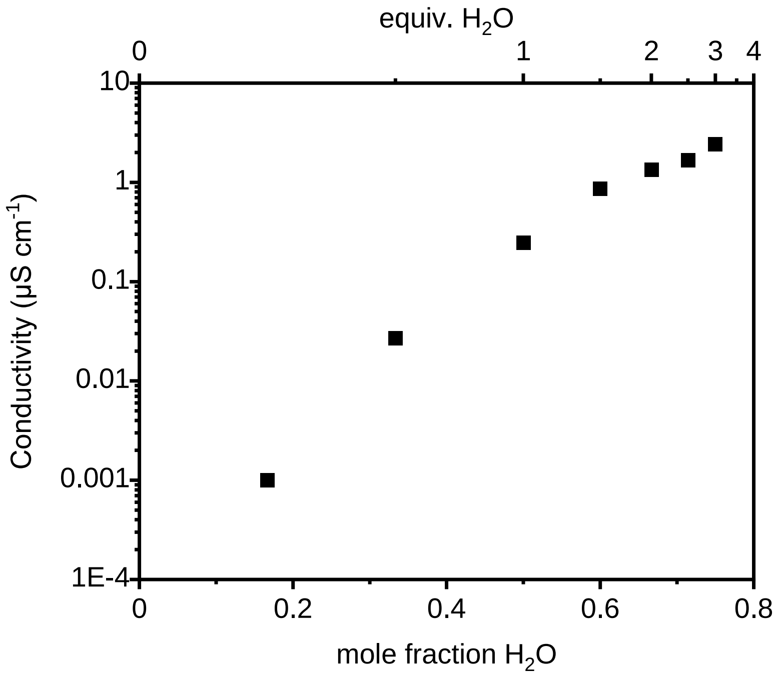

Fig. S10. Conductivity of C_{10}-TMG (ordinate) and amount of H_2O added.

8. Extended discussion of quantification method

Choice of method

In direct air capture experiments, carbon dioxide and water are co-absorbed by the C_n-TMG bases. For this reason, simple gravimetric measurements cannot be used to quantify CO_2 uptake, as it overestimates the capacity by not distinguishing between mass gain originating from CO_2 and water. The total alkalinity of the sample does not change upon CO_2 capture, and for this reason manual acid/base titrations are unsuitable for CO_2 uptake determination. Instead, we opted to use ^{13}C NMR spectroscopy to quantify the ratio between HCO_3^- and CO_3^{2-} and determine the CO_2 uptake from a standard curve. Alternatively, pH measurements can be used given that a standard curve is first produced. In this procedure, the sample is dissolved in D_2O after a direct air capture experiment and subsequently analyzed by NMR spectroscopy.

Quantification of CO_2 uptake by aprotic bases using NMR spectroscopy

The following method and associated discussions are only relevant for aprotic bases – bases that do not form CO_2 adducts (such as carbamates).

In water, HCO_3^- and CO_3^{2-} exist in rapid equilibrium faster than the NMR time-scale. For this reason, only one ^{13}C resonance is observed in a system containing both species. Figure S11 depicts the ^{13}C chemical shift (abscissa) of HCO_3^-/ CO_3^{2-} and the fraction of HCO_3^- (ordinate). *Note: the contribution of dissolved CO_2 is negligible in the relevant pH range and carbonate-ion concentration.*

If we define molar loading as:

$$\alpha = \frac{n(CO_2)}{n(base)} \text{ (eq. 2)}$$

It follows that when $0 < \alpha < 0.5$, captured CO_2 speciates almost exclusively as CO_3^{2-} (*i*) whereas when $0.5 < \alpha < 1$, captured CO_2 speciates as HCO_3^- and CO_3^{2-} (*ii*) until $\alpha \sim 1$, where CO_2 speciates predominantly as HCO_3^- (these statements assume that the base reacting with CO_2 is strong ($pK_{aH} \sim 14$) and the concentration of the base is not diluted to the mM range). Given that the ^{13}C NMR chemical shift of HCO_3^-/ CO_3^{2-} depends on the ratio between HCO_3^- and CO_3^{2-}, the chemical shift can be used to determine α within the range $0.5 < \alpha < 1$.

$$2\ Base + CO_2 + H_2O \rightarrow 2[BaseH]^+ + CO_3^{2-}, 0 < \alpha < 0.5\ (i)$$

$$CO_3^{2-} + CO_2 + H_2O \rightarrow 2HCO_3^-, 0.5 < \alpha < 1\ (ii)$$

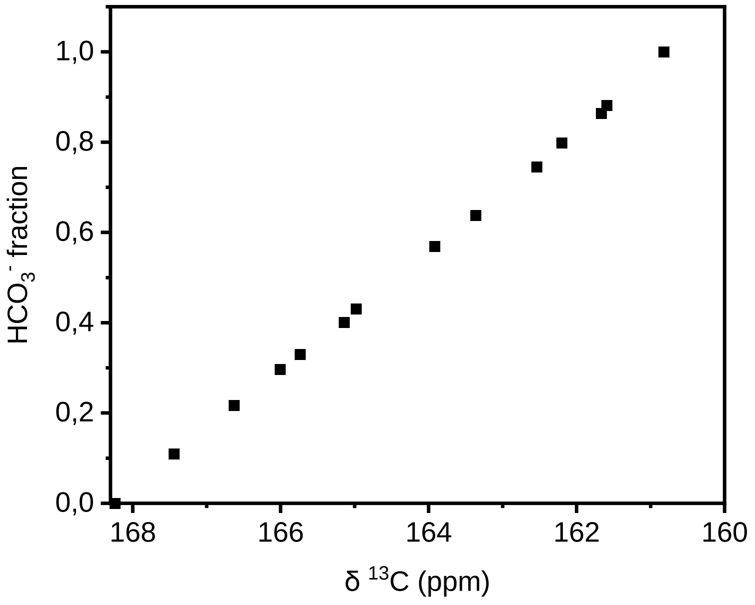

Fig. S11. ^{13}C NMR (D_2O) chemical shift (abscissa) of HCO_3^-/CO_3^{2-} depicted as a mole fraction of HCO_3^- (ordinate). Spectra were prepared by dissolving $KHCO_3$ and K_2CO_3 in D_2O at different ratios.[45]

In the range $0 < \alpha < 0.5$, the captured CO_2 speciates almost exclusively as CO_3^{2-} independent of α. Hence, ^{13}C NMR chemical shift cannot be used to determine α in this range. The CO_2 loading can however be determined by spiking the unknown sample (with loading $\alpha = \alpha_U$) with a standard sample of known loading α_S. When the known sample is added to the unknown sample, the total loading α_{tot} is the weighted average of α_S and α_U. The contribution to the weighted average by each sample is the mole fraction of each component x_S and x_U respectively (eq. 3-5).

$$\alpha_{tot} = \alpha_S * x_S + \alpha_U * x_U, 0 < x_S < 1 \text{ (eq. 3)}$$

$$x_S = \frac{n_S}{n_U + n_S}, x_U = \frac{n_U}{n_U + n_S} \text{ (eq. 4)}$$

$$x_S + x_U = 1 \Leftrightarrow x_U = 1 - x_S \text{ (eq. 5)}$$

Where n_i is the moles of absorbent originating from the *i*th sample. Inserting into eq. 3 gives:

$$\alpha_{tot} = \alpha_S * x_S + \alpha_U * (1 - x_s), 0 < x_S < 1 \text{ (eq. 6)}$$

Which simplifies to the following linear relationship (eq. 7) between α_{tot} and x_S. The intercept is the loading of the unknown α_U:

$$\alpha_{tot} = (\alpha_S - \alpha_U) * x_S + \alpha_U \text{ (eq. 7)}$$

The method is most accurate when α_U is close to 0.5.

Quantification of water uptake

After a direct air capture experiment, the mass of the sample increases due to uptake of water and CO_2 by *m*(H_2O) and *m*(CO_2) respectively (eq. 8):

$$\Delta m = m(H_2O) + m(CO_2) \text{ (eq. 8)}$$

The value *m*(CO_2) was calculated from the CO_2 loading α determined by chemical shift analysis:

$$m(CO_2) = n(CO_2) * M(CO_2) = \alpha * n(C_n\text{-}TMG) * M(CO_2) \text{ (eq. 9)}$$

The water uptake in *wt%* was calculated by ratio of *m*(H_2O) and *m*(C_n-TMG):

$$H_2O\ (wt\%) = \frac{m(H_2O)}{m(C_n\text{-}TMG)} * 100\% = \frac{\Delta m - m(CO_2)}{m(C_n\text{-}TMG)} * 100\% \text{ (eq. 10)}$$

Inserting (eq. 9) into (eq. 10) gives:

$$H_2O\ (wt\%) = \frac{\Delta m - \alpha * n(C_n\text{-}TMG) * M(CO_2)}{m(C_n\text{-}TMG)} * 100\% \text{ (eq. 11)}$$

9. Standard curve for CO_2 loading determination by ^{13}C NMR Spectroscopy

Standard curve: Standard curve was prepared by addition of CO_2-rich and CO_2-poor C_n-TMG/ethylene glycol standard solutions in different ratios and dilution by addition of D_2O affording a desired molal concentration of 0.090 mol kg^{-1}. The standard curve was prepared in duplicate using C_{10}-TMG as absorbent and replicated once more using C_7-TMG as absorbent. Pipetting aqueous solutions of C_n-TMG was found to be inaccurate. For this reason, all quantities are reported as masses. The results are depicted in figure S12.

CO_2-rich standard solution. To a 20 mL vial, C_{10}-TMG (0.5000 g, 1.96 mmol) and ethylene glycol (0.0924 g, 1.49 mmol, 0.76 equiv.) was added, as well as a magnetic stir bar. The vial was sealed with a screw-cap containing a septum and the total mass was recorded. The absorbent and ethylene glycol mixture was briefly stirred to homogenize, where after the system was purged for 15 seconds with CO_2 supplied by a freshly prepared double-wrapped balloon at ambient temperature. After purging, the vial was tightly sealed and left to stir (100 rpm) at ambient temperature under CO_2 atmosphere. After stirring for 2 hours, the balloon was removed, and the atmosphere was exchanged back to air. The mass was immediately recorded which corresponded to a CO_2 loading of 0.84 mol(CO_2):mol(C_{10}-TMG). The mixture was diluted by addition of D_2O (10.5762 g) affording C_{10}-TMG at a molal concentration of 0.182 mol kg^{-1}.

CO_2-poor standard solution. Since the absorbents are water insoluble in the CO_2-free state, it was necessary to partially load the CO_2-poor standard solution to ensure complete water solubility. This was achieved by the mixing CO_2-free C_{10}-TMG with the CO_2-rich C_{10}-TMG according to the following procedure: To a 20 mL vial, C_{10}-TMG (0.1843 g, 0.721 mmol) was added as well as 3.5307 g of CO_2-rich standard solution. The mixture was diluted by addition of D_2O (4.0087 g) affording C_{10}-TMG at a molal concentration of 0.181 mol kg^{-1} and a CO_2 loading of 0.40 mol(CO_2):mol(C_n-TMG).

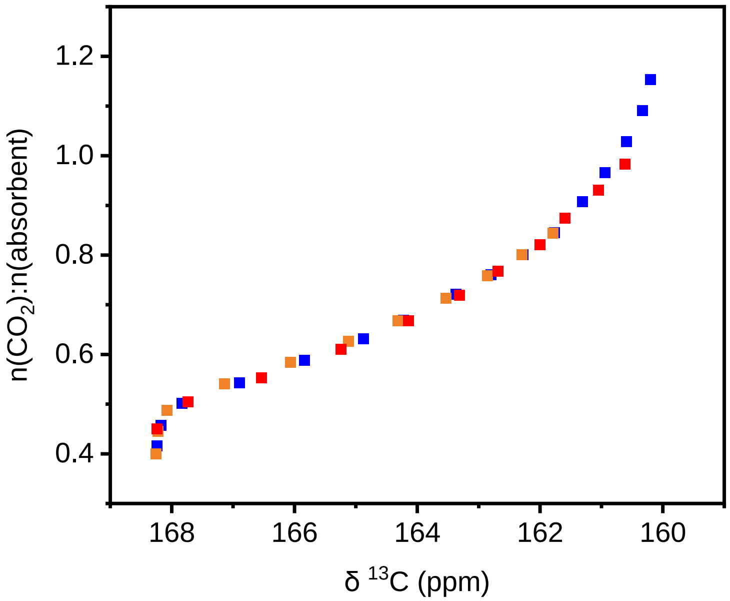

Fig. S12. ^{13}C NMR (126 MHz, D_2O) chemical shift of HCO_3^-/CO_3^{2-} (abscissa) and molar CO_2 loading of C_n-TMG (ordinate). Red (C_7-TMG), orange (C_{10}-TMG), blue (C_{10}-TMG). Non-linear standard curve (not shown) was obtained by polynomial fitting (n = 4) of data in the range 168 ppm > δ > 160.5 ppm.

10. Graphical representation of experimental setup for humidity regulated capture experiments

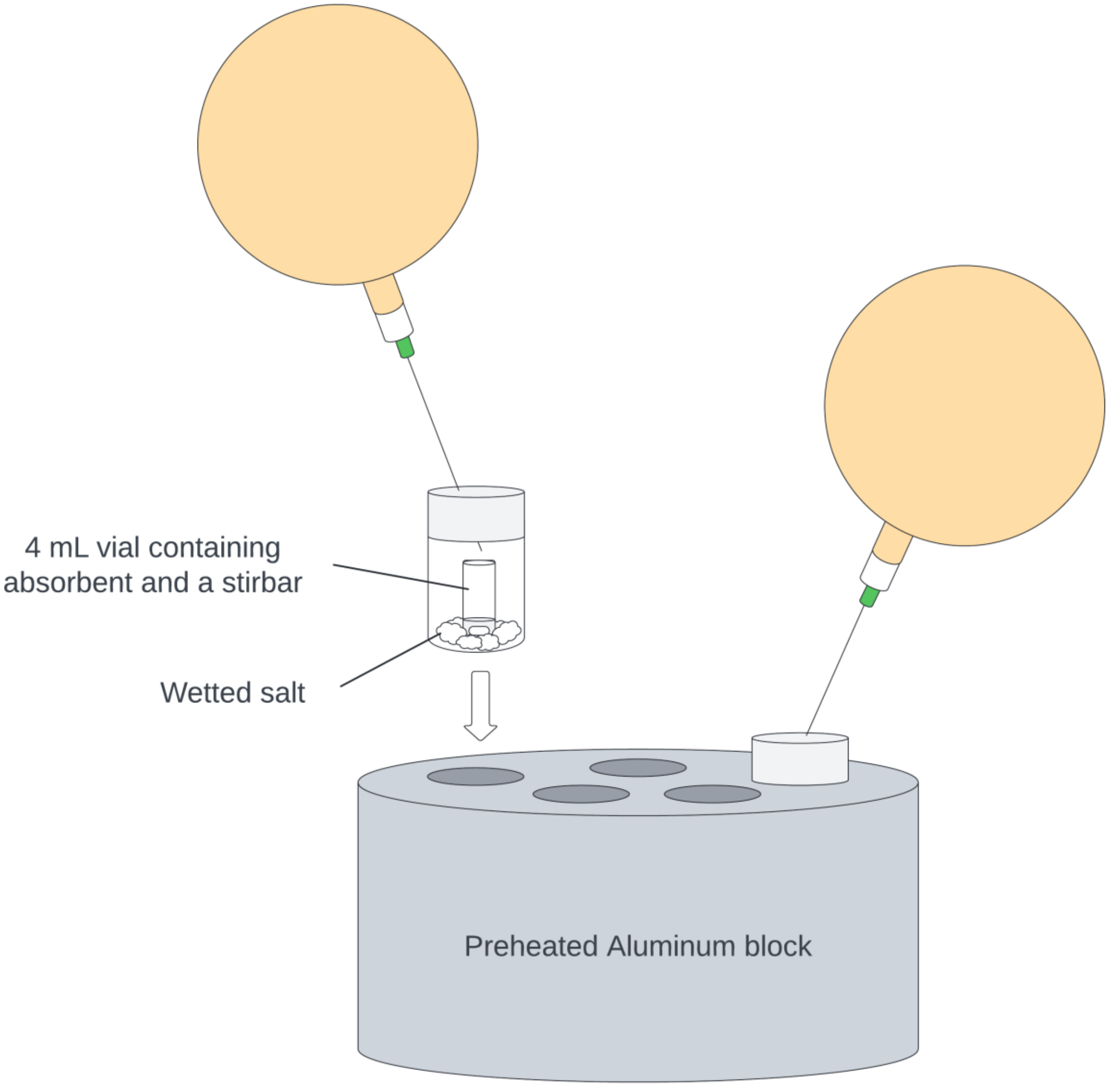

Fig. S13. Graphical representation of experimental setup for humidity regulated capture experiments. The tops of the vials were wrapped in cotton and foil (not shown).

11. NMR Spectra of absorbents

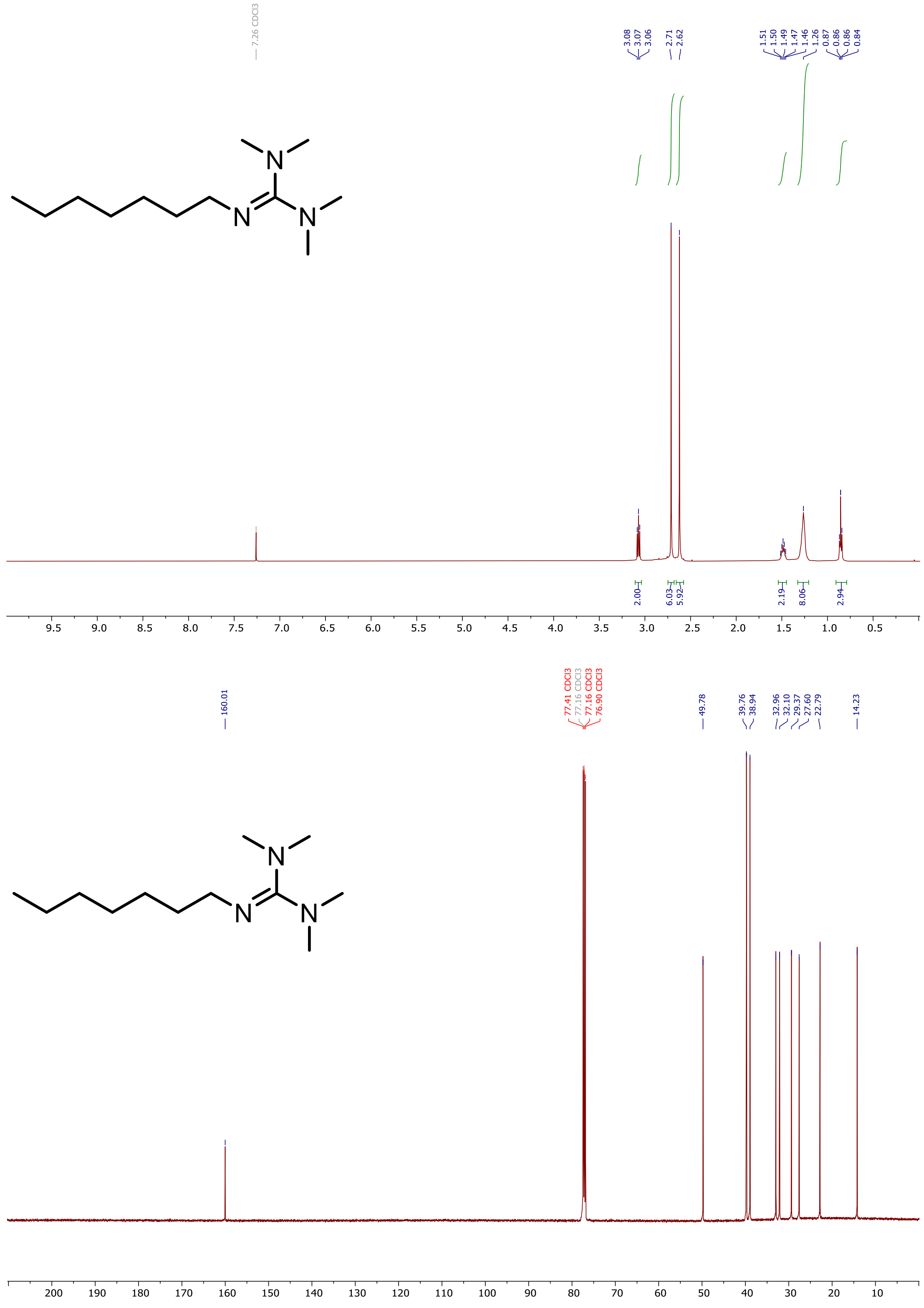

Fig. S14. ^1H and ^{13}C NMR ($CDCl_3$) spectra of C_7-TMG.

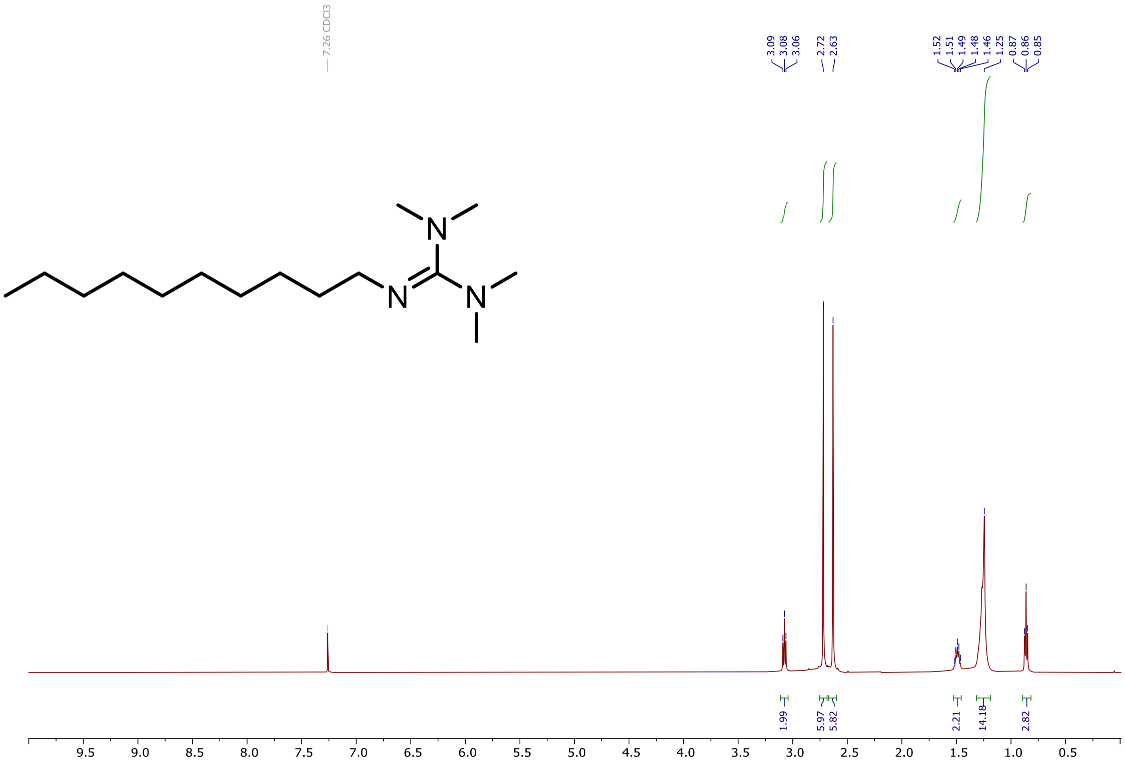

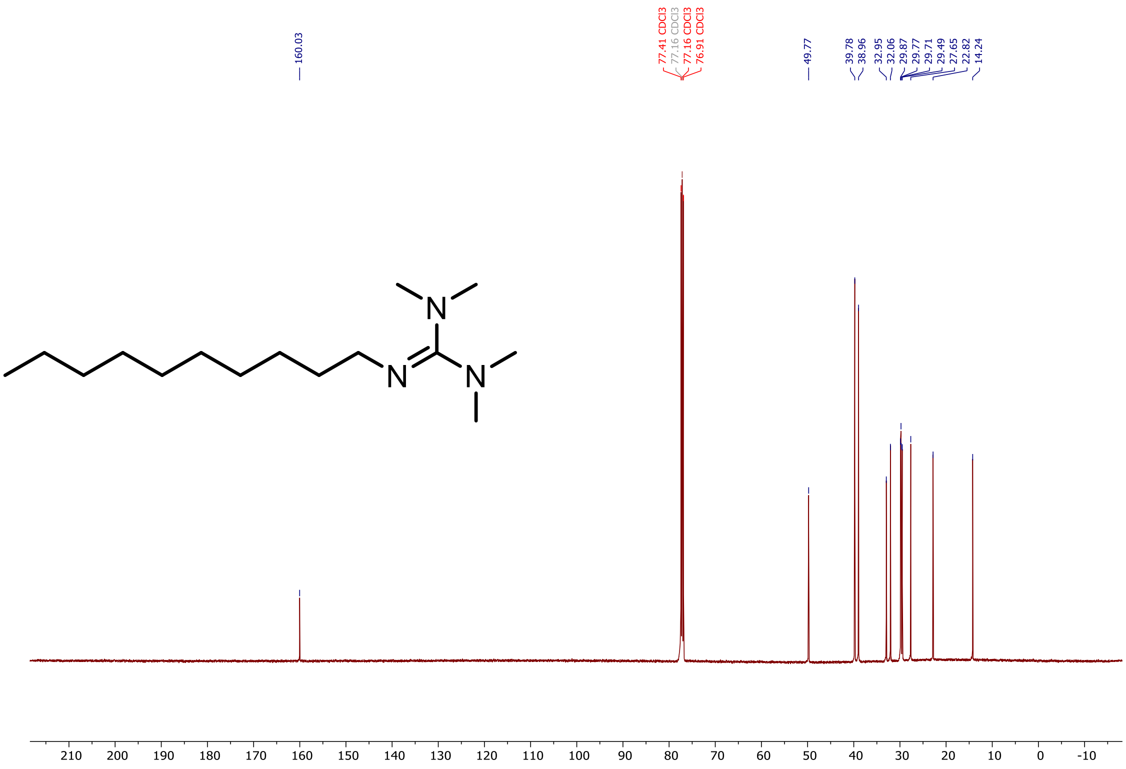

Fig. S15. ^{1}H and ^{13}C NMR ($CDCl_3$) spectra of C_{10}-TMG.

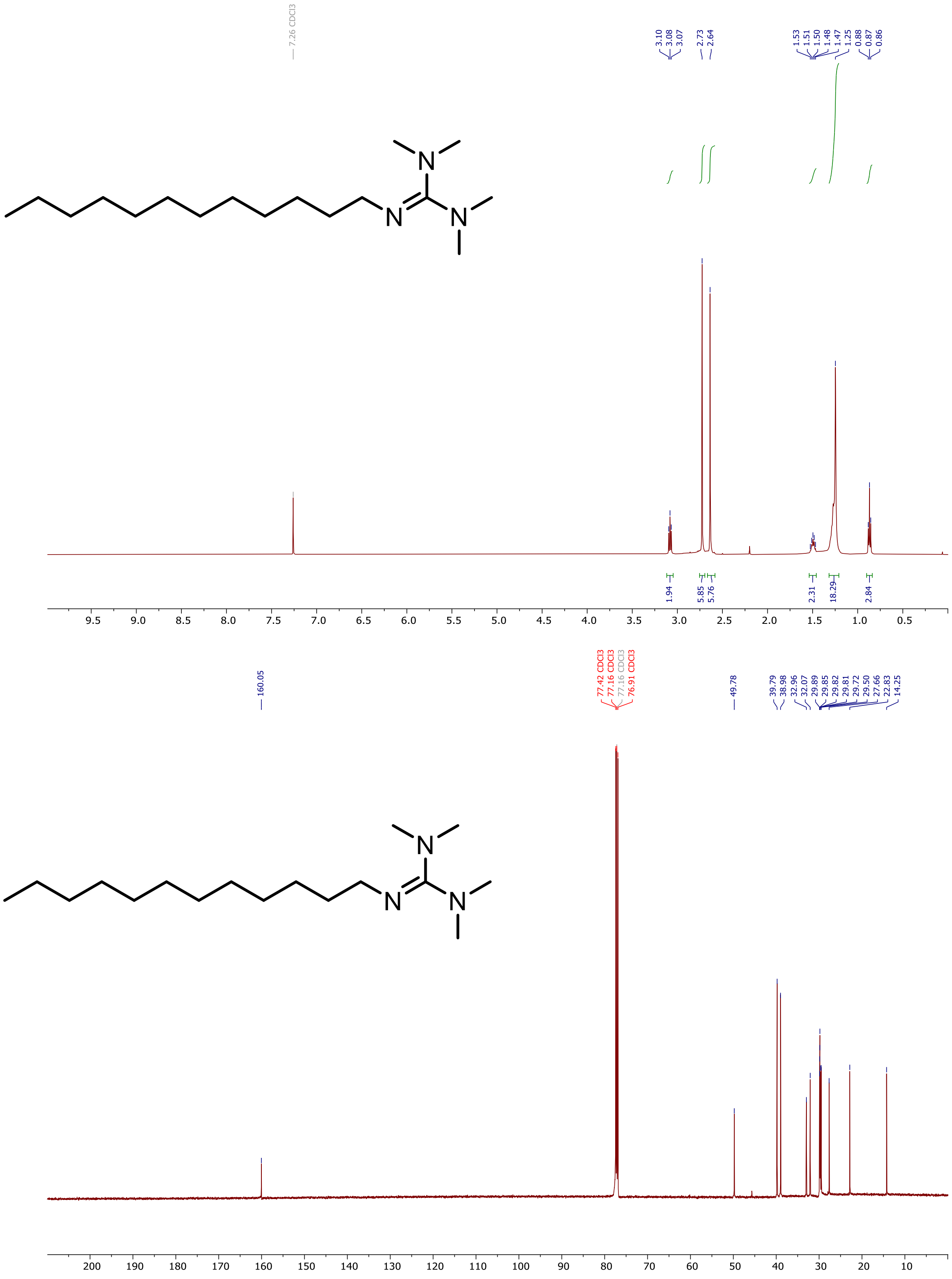

Fig. S16. ^{1}H and ^{13}C NMR ($CDCl_3$) spectra of C_{12}-TMG.

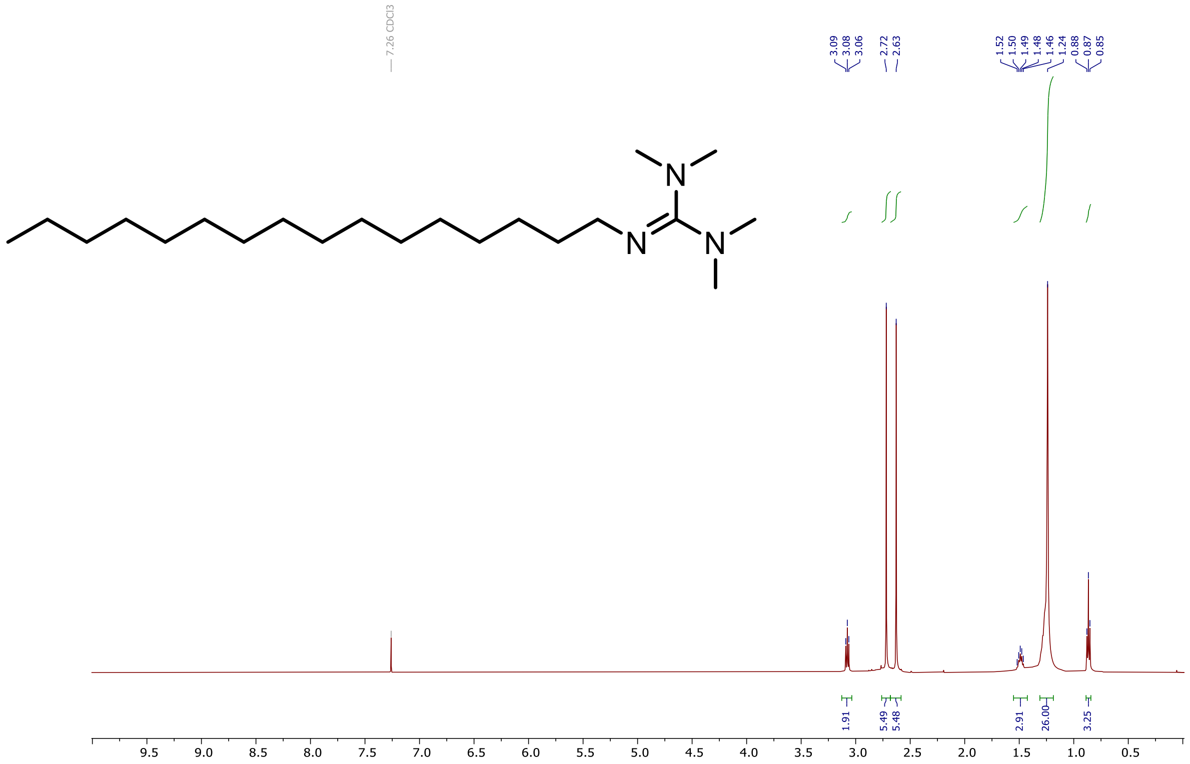

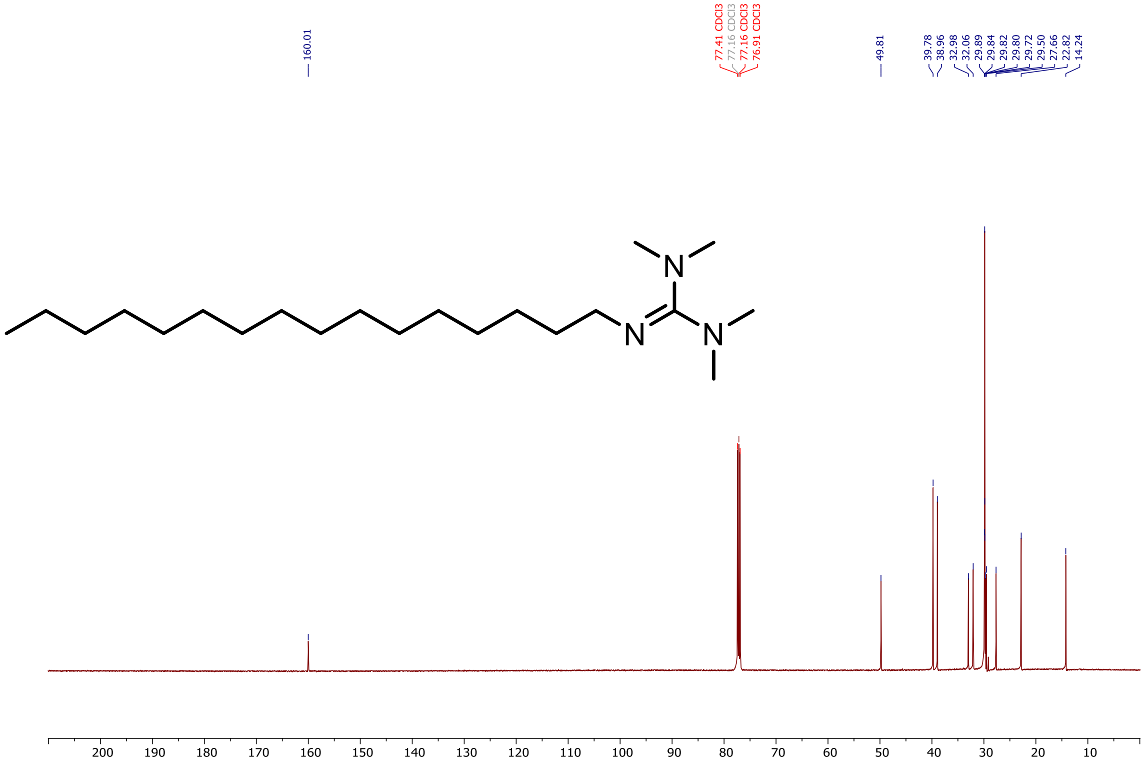

Fig. S17. ^{1}H and ^{13}C NMR ($CDCl_3$) spectra of C_{16}-TMG.

12. Infrared Spectra of absorbents

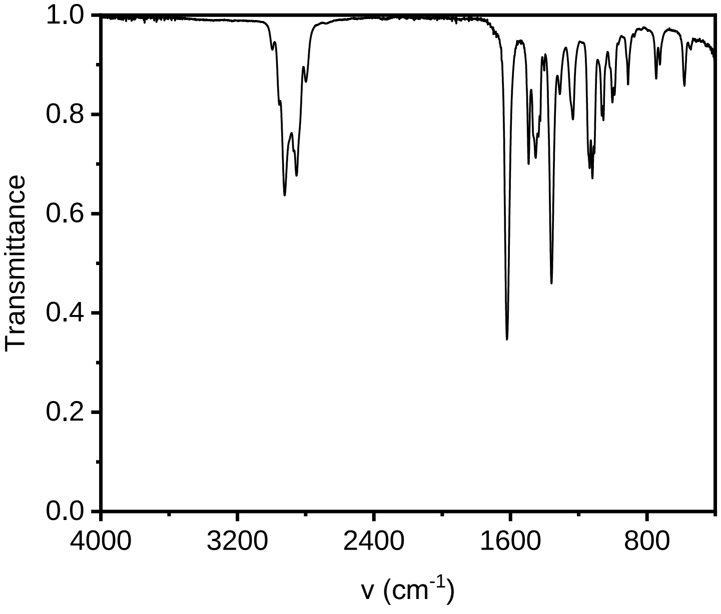

Fig. S18. IR Spectrum (neat) of C_7-TMG

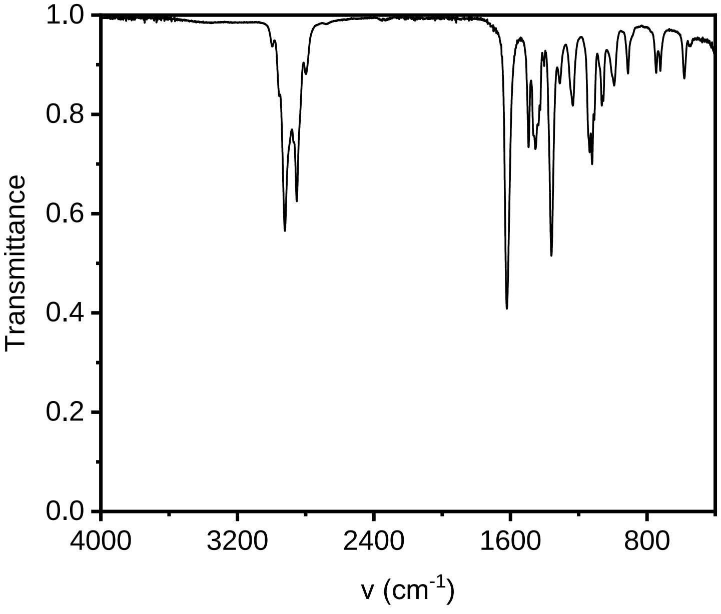

Fig. S19. IR Spectrum (neat) of C_{10}-TMG

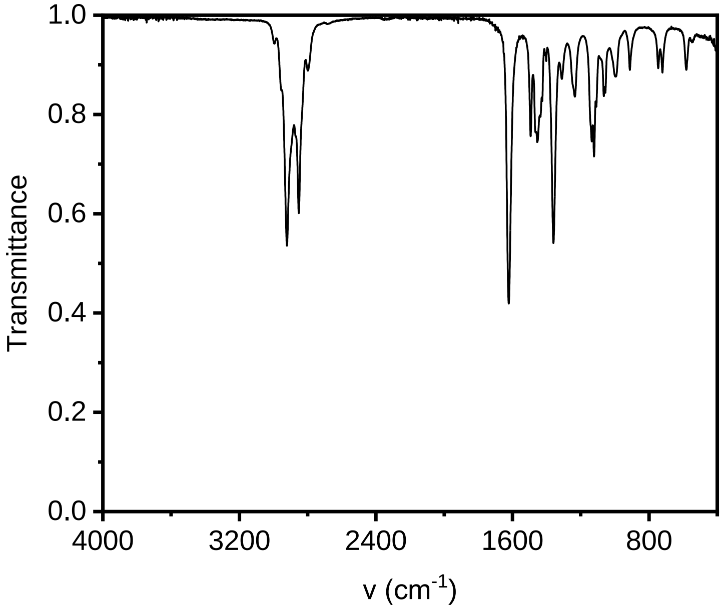

Fig. S20. IR Spectrum (neat) of C_{12}-TMG

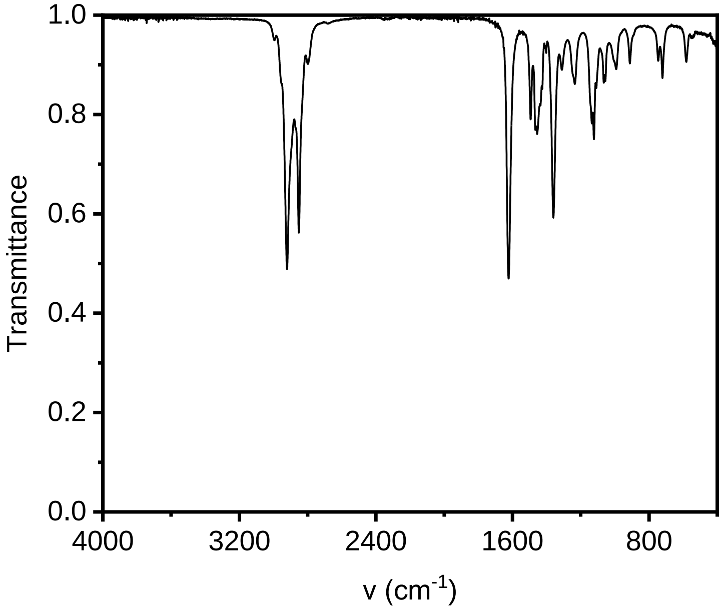

Fig. S21. IR Spectrum (neat) of C_{16}-TMG

13. ^{13}C NMR spectra of CO_2-loaded absorbents

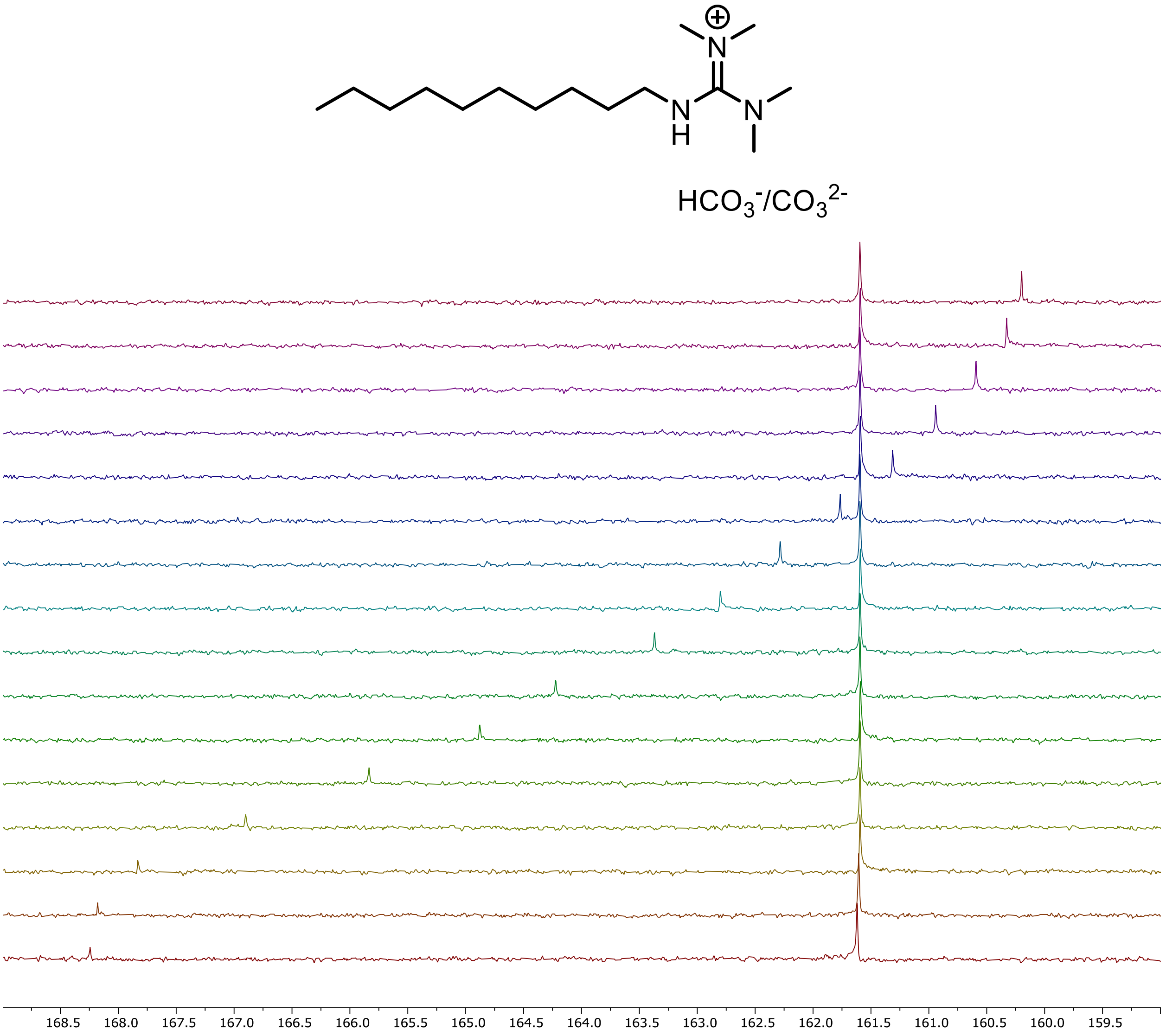

Fig. S22. ^{1}H and ^{13}C NMR (126 MHz, D_2O) spectra of C_{10}-TMG loaded with various amounts of CO_2. Resonance at 161.59 ppm corresponds to guanidinium carbon.

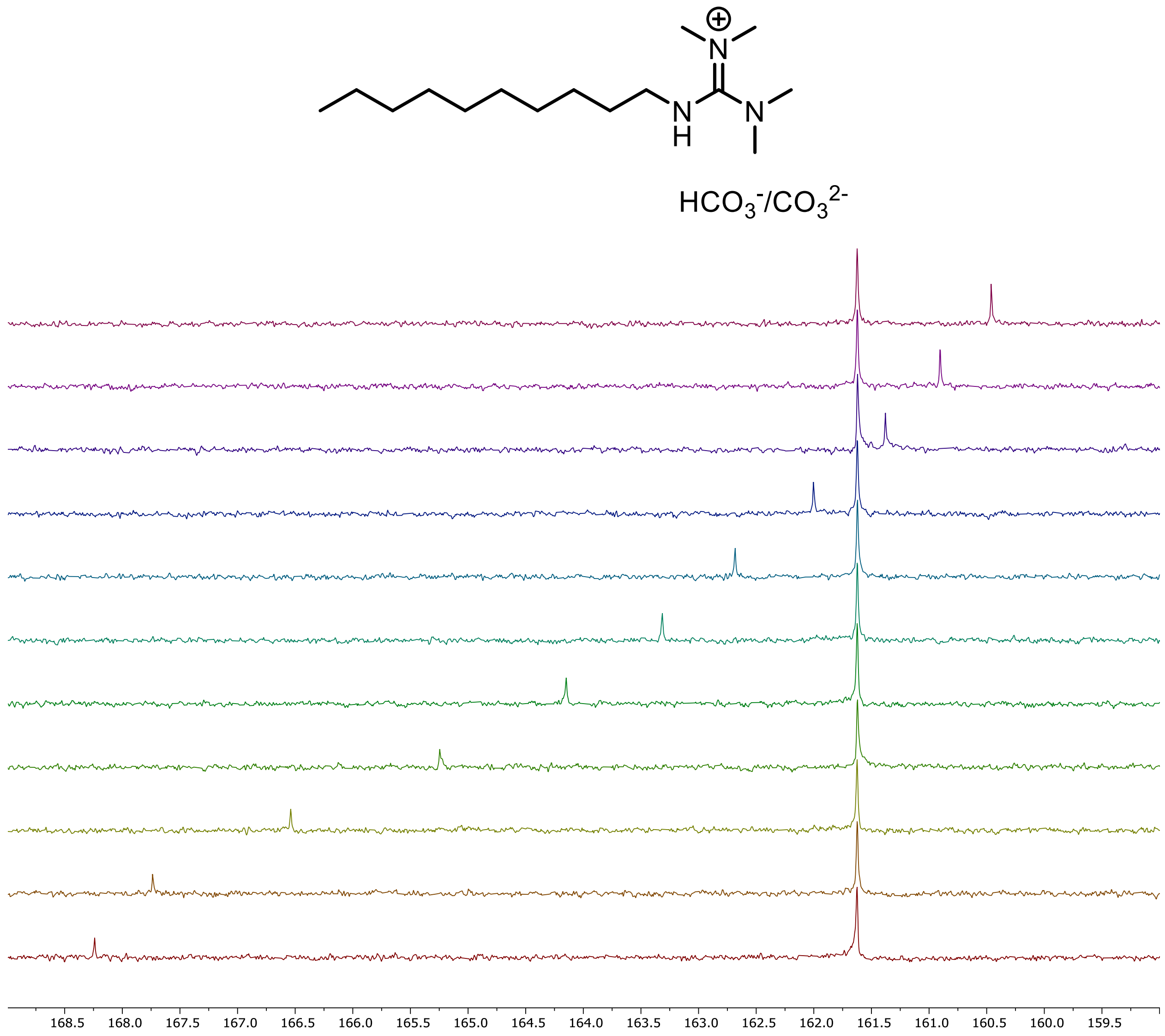

Fig. S23. ^{1}H and ^{13}C NMR (126 MHz, D_2O) spectra of C_{10}-TMG loaded with various amounts of CO_2. Resonance at 161.59 ppm corresponds to guanidinium carbon.

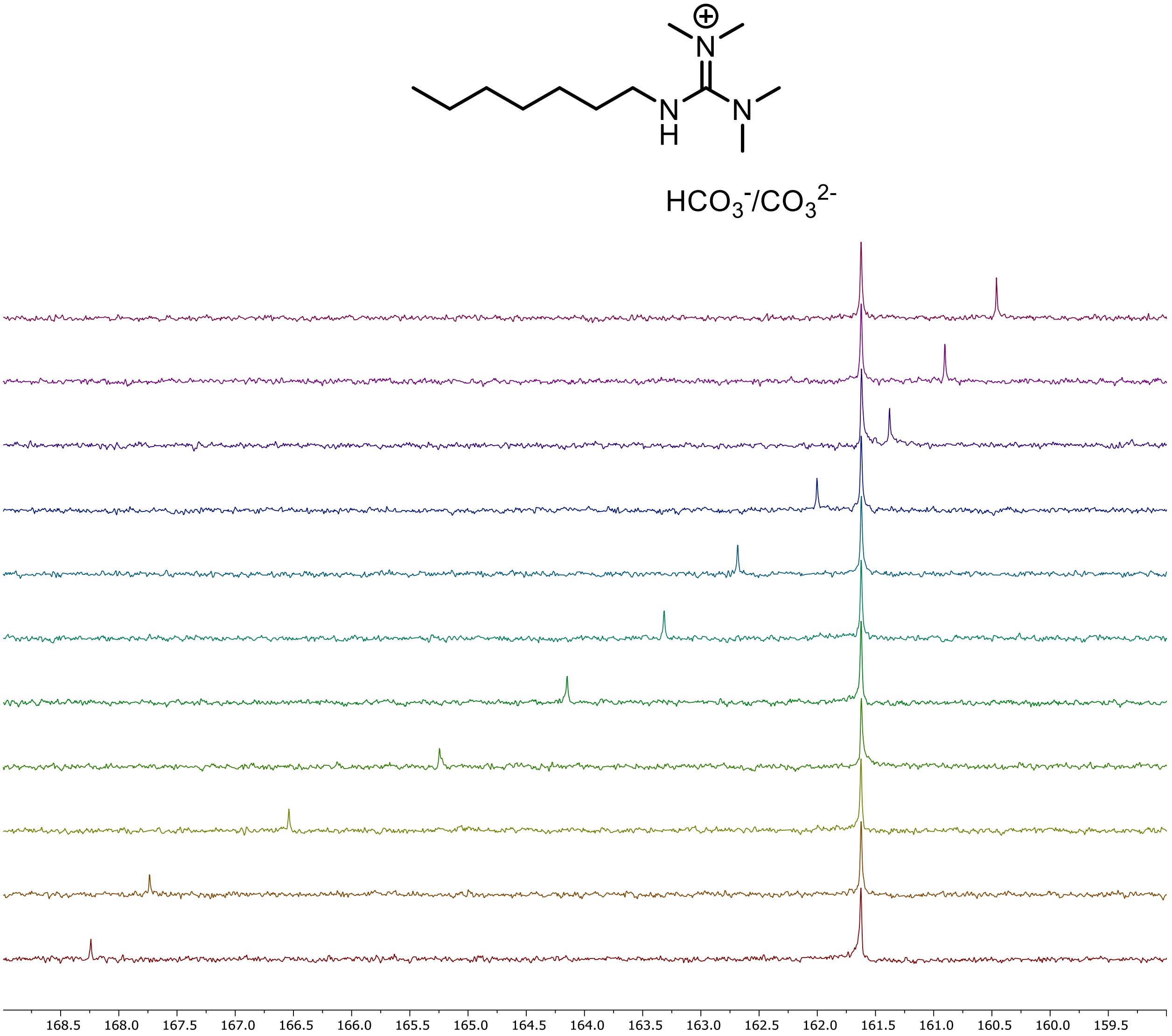

Fig. S24. ^{1}H and ^{13}C NMR (126 MHz, D_2O) spectra of C_7-TMG loaded with various amounts of CO_2. Resonance at 161.62 ppm corresponds to guanidinium carbon.

14. Raw kinetic data from Direct Air Capture experiments

Table S1. Raw data from direct air capture experiments. *Exposure* refers to moles of CO_2 (from air) passed over the sample divided by the mass of the sample. The vials were measured while *empty*, *before capture* and *post capture*. The ^{13}C chemical shift refers to the chemical shift (126 MHz) of HCO_3^-/CO_3^{2-} obtained after dissolving the sample in D_2O. Samples that captured below half capacity are denoted with "Sample was spiked".

		Exposure	Empty	Before capture	Post capture	^{13}C NMR chemical shift
**Compound**	**RH%**	CO_2 (mmol:g)	m(vial) (g)	m(vial) (g)	m(vial) (g)	HCO_3^-/CO_3^{2-} (ppm)
C_7-TMG	58	7.12	7.7326	7.7822	7.7996	167.15
C_7-TMG	58	10.17	7.8141	7.8652	N/A	161.63
C_7-TMG	58	20.75	7.6944	7.7445	7.7714	160.92
C_7-TMG	64	5.88	7.8815	7.9315	7.9502	167.79
C_7-TMG	64	8.73	7.6584	7.7087	7.7349	162.57
C_7-TMG	64	10.81	7.7536	7.8017	7.8258	161.35
C_7-TMG	64	14.29	7.8725	7.9231	7.954	160.97
C_7-TMG	64	20.92	7.6254	7.6751	7.705	160.95
C_7-TMG	75	5.88	7.8607	7.9114	7.9456	164.11
C_7-TMG	75	5.88	7.8455	7.8959	7.9284	163.93
C_7-TMG	75	5.88	7.8447	7.8949	7.926	164.32
C_7-TMG	75	10.31	7.7753	7.8258	7.8622	161.51
C_7-TMG	75	20.58	7.7987	7.849	7.891	161.59
C_7-TMG	84	4.18	7.801	7.8509	7.8925	167.50
C_7-TMG	84	5.88	7.7239	7.7726	7.8158	163.42
C_7-TMG	84	5.91	7.9687	8.0185	8.0626	163.32
C_7-TMG	84	11.50	7.7255	7.7707	7.818	162.31
C_7-TMG	84	20.58	7.779	7.8286	7.8817	162.45
C_7-TMG	100	4.18	7.8094	7.8588	N/A	166.38
C_7-TMG	100	5.88	7.6719	7.7213	7.7787	163.50
C_7-TMG	100	11.01	7.7462	7.7934	7.8586	163.24
C_{10}-TMG	58	9.50	7.8882	7.9429	7.9638	164.20
C_{10}-TMG	58	20.87	7.7157	7.7655	7.7876	161.61
C_{10}-TMG	64	7.46	7.7146	7.7644	7.7803	167.08
C_{10}-TMG	64	9.61	7.7527	7.8068	7.8344	162.35
C_{10}-TMG	64	10.27	7.7766	7.8272	7.8485	163.07
C_{10}-TMG	64	14.82	7.8208	7.8703	7.8729	161.44
C_{10}-TMG	64	20.87	7.7215	7.7713	7.7953	161.44
C_{10}-TMG	75	2.94	7.8572	7.9066	7.9223	Sample was spiked
C_{10}-TMG	75	5.88	7.8763	7.9258	7.9531	164.80
C_{10}-TMG	75	5.88	7.7997	7.8501	7.8771	164.96
C_{10}-TMG	75	5.90	7.8252	7.8748	7.9013	165.02
C_{10}-TMG	75	8.82	7.8769	7.9267	7.958	162.42
C_{10}-TMG	75	10.78	7.7740	7.8222	7.8557	161.96
C_{10}-TMG	75	10.35	7.7995	7.8497	7.883	162.02
C_{10}-TMG	75	20.58	7.7712	7.8219	7.8546	161.82
C_{10}-TMG	84	4.43	7.7664	7.8167	7.8465	166.86

C_{10}-TMG	84	5.88	7.7539	7.8037	7.8395	163.97
C_{10}-TMG	84	5.88	7.6997	7.7497	7.7852	164.06
C_{10}-TMG	84	5.88	7.6962	7.7464	7.7818	164.04
C_{10}-TMG	84	9.84	7.8308	7.8836	7.9289	162.40
C_{10}-TMG	84	20.58	7.6896	7.7398	7.7792	162.43
C_{10}-TMG	100	4.42	7.7816	7.8318	7.8717	166.17
C_{10}-TMG	100	5.88	7.8002	7.8504	7.8966	163.76
C_{10}-TMG	100	10.74	7.8389	7.8873	7.9494	163.62
C_{10}-TMG	100	10.31	7.7791	7.8295	7.8883	163.49
C_{12}-TMG	58	10.67	7.7931	7.8418	7.8512	Sample was spiked
C_{12}-TMG	58	20.83	7.6994	7.7493	7.7585	165.51
C_{12}-TMG	64	10.83	7.7769	7.8249	7.8391	167.56
C_{12}-TMG	64	16.23	7.8801	7.9298	7.9453	162.66
C_{12}-TMG	64	20.54	7.7622	7.8128	7.8333	162.49
C_{12}-TMG	75	2.94	7.7988	7.8489	7.8594	Sample was spiked
C_{12}-TMG	75	5.88	7.8163	7.8666	7.8872	167.7637
C_{12}-TMG	75	5.88	7.7845	7.8348	N/A	168.1749
C_{12}-TMG	75	5.88	7.8132	7.8628	N/A	168.0518
C_{12}-TMG	75	8.82	7.8296	7.88	7.906	164.0691
C_{12}-TMG	75	9.73	7.788	7.8414	7.8708	163.54
C_{12}-TMG	75	10.46	7.8125	7.8622	7.89	162.34
C_{12}-TMG	75	20.58	7.7457	7.7959	7.824	161.91
C_{12}-TMG	84	4.75	7.8433	7.8938	7.9187	167.88
C_{12}-TMG	84	5.88	7.7029	7.7533	7.7822	165.72
C_{12}-TMG	84	9.96	7.755	7.8072	7.8449	162.70
C_{12}-TMG	84	20.58	7.7272	7.777	7.8113	162.60
C_{12}-TMG	100	4.59	7.8436	7.894	7.9344	166.51
C_{12}-TMG	100	5.88	7.7572	7.8072	7.8468	164.80
C_{12}-TMG	100	9.98	7.7988	7.8509	7.9029	163.47
C_{12}-TMG	100	10.50	7.8262	7.8757	7.9222	163.37
C_{12}-TMG	100	12.27	7.8577	7.9081	7.9619	163.84
C_{16}-TMG	64	9.61	7.765	7.8191	7.8229	Sample was spiked
C_{16}-TMG	75	2.94	7.8771	7.9273	7.9339	Sample was spiked
C_{16}-TMG	75	5.89	7.8535	7.9037	7.9157	Sample was spiked
C_{16}-TMG	75	5.88	7.7696	7.8187	7.8309	Sample was spiked
C_{16}-TMG	75	8.82	7.7946	7.8437	7.8626	165.96
C_{16}-TMG	75	10.13	7.9071	7.9584	7.9778	164.18
C_{16}-TMG	75	10.31	7.8505	7.9009	7.9199	164.59
C_{16}-TMG	75	20.58	7.6472	7.6967	7.7178	162.33
C_{16}-TMG	84	5.88	7.6969	7.7462	7.7693	166.32
C_{16}-TMG	84	9.42	7.8452	7.9004	7.934	163.32
C_{16}-TMG	100	5.88	7.6569	7.7062	7.7348	163.96
C_{16}-TMG	100	9.25	7.7912	7.8474	7.8846	163.24
C_{16}-TMG	100	9.86	7.8666	7.9193	7.9516	163.26
